# Autophagy facilitates intracellular survival of pathogenic rickettsiae in macrophages via evasion of autophagosomal maturation and reduction of microbicidal pro-inflammatory IL-1 cytokine responses

**DOI:** 10.1128/spectrum.02791-23

**Published:** 2023-10-11

**Authors:** Oliver H. Voss, Hodalis Gaytan, Saif Ullah, Mohammad Sadik, Imran Moin, M. Sayeedur Rahman, Abdu F. Azad

**Affiliations:** 1 Department of Microbiology and Immunology, University of Maryland School of Medicine, Baltimore, Maryland, USA; Texas A&M University, College Station, Texas, USA

**Keywords:** *R. typhi*, *R. rickettsii*, *R. montanensis*, autophagy, IL-1α, IL-1β, macrophages, bacterial host dissemination

## Abstract

The genus *Rickettsia* is comprised of obligate intracellular bacterial parasites of a wide range of arthropod and vertebrate hosts. Some *Rickettsia* species (spp.) are responsible for serious human diseases globally. One interesting feature of these stealthy group of pathogens is their ability to exploit host cytosolic defense responses to their benefits. However, the precise mechanism by which pathogenic *Rickettsia* spp. elude host immune defense responses remains to be determined. Here, we observed that pathogenic *Rickettsia typhi* and *Rickettsia rickettsii* (Sheila Smith [SS]), but not non-pathogenic *Rickettsia montanensis*, become ubiquitinated and induce autophagy upon entry into bone marrow-derived macrophages. Moreover, unlike *R. montanensis*, both *R. typhi* and *R. rickettsii* (SS) colocalized with LC3B and not with Lamp2 upon host cell entry. Finally, we observed that both *R. typhi* and *R. rickettsii*, but not *R. montanensis*, reduce pro-inflammatory interleukin-1 (IL-1) cytokine responses, likely via an autophagy-mediated mechanism. In summary, we identified a previously unappreciated pathway by which both pathogenic *R. typhi* and *R. rickettsii* (SS), but not the non-pathogenic *R. montanensis,* become ubiquitinated, induce autophagy, avoid autolysosomal destruction, and reduce microbicidal IL-1 cytokine responses to establish an intracytosolic niche in macrophages.

**IMPORTANCE:**

*Rickettsia* spp. are intracellular bacterial parasites of a wide range of arthropod and vertebrate hosts. Some rickettsiae are responsible for several severe human diseases globally. One interesting feature of these pathogens is their ability to exploit host cytosolic defense responses to their benefits. However, the precise mechanism by which pathogenic *Rickettsia* spp. elude host defense responses remains unclear. Here, we observed that pathogenic *Rickettsia typhi* and *Rickettsia rickettsii* (Sheila Smith [SS]), but not non-pathogenic *Rickettsia montanensis*, become ubiquitinated and induce autophagy upon entry into macrophages. Moreover, unlike *R. montanensis*, *R. typhi* and *R. rickettsii* (SS) colocalized with LC3B but not with Lamp2 upon host cell entry. Finally, we observed that both *R. typhi* and *R. rickettsii* (SS), but not *R. montanensis*, reduce pro-inflammatory interleukin-1 (IL-1) responses, likely via an autophagy-mediated mechanism. In summary, we identified a previously unappreciated pathway by which both pathogenic *R. typhi* and *R. rickettsii* (SS) become ubiquitinated, induce autophagy, avoid autolysosomal destruction, and reduce microbicidal IL-1 cytokine responses to establish an intracytosolic niche in macrophages.

## INTRODUCTION

A plethora of intracytosolic bacteria utilize sophisticated strategies to circumvent host defense responses to promote their survival within the host (1, 2). Rickettsiae are arthropod-borne obligate intracellular bacteria with both symbiotic and pathogenic lifestyles. *Rickettsia prowazekii*, *Rickettsia typhi*, *Rickettsia rickettsii* (Sheila Smith [SS]), and *Rickettsia conorii*, etiological agents for epidemic typhus, murine typhus, Rocky Mountain spotted fever, and Boutonneuse fever, respectively, are well-known human pathogens (3, 4). Apart from their historical record, the global impact of rickettsial infections is illustrated by the resurgence of known, as well as the rise of newly identified rickettsial pathogens (5). Infections of humans with *R. rickettsii* (SS) continue to cause severe public health threats in South and Central America (6). The resurgence of *R. conorii* in Europe, the Middle East, and Africa further highlights current threats of rickettsial diseases globally (7). In addition, arthropod-borne rickettsial diseases are also on the rise in the USA, as exemplified by recent outbreaks of *R. rickettsii* (SS) in Arizona (8) and of *R. typhi* in California (9) and Texas (10).

Entry into eukaryotic host cells, followed by intracytosolic growth and dissemination to neighboring cells has long been considered as a conserved process of *Rickettsia* spp. However, it is important to note (and often overlook) that many described rickettsiae are naturally maintained in arthropods and are considered non-pathogenic, as they cause no or a very mild disease in humans (3, 11, 12). *Rickettsia* spp. are introduced into the host’s dermis by infected arthropods and the first host defense cells encountered are macrophages and dendritic cells (4). In particular, macrophages are crucial in either terminating an infection at an early stage or succumbing to pathogen colonization, thereby contributing to the dissemination of *Rickettsia* spp. to distant organs of the host (4). Upon host cell entry, rickettsiae encounter cytosolic host defense responses initiated after sensing the bacteria or associated danger signals. Commonly invading bacteria encounter autophagy and inflammasome responses, two functionally interconnected pathways (13, 14), which typically provide the appropriate defense measures against intracellular pathogens (15, 16). Importantly, recent reports suggest that autophagy acts on intracellular microbes upstream of the inflammasome (17 – 19). Autophagy is triggered by ubiquitination of the intracellular bacterium (20). Ubiquitin (Ub) coats the bacterial surface and recruits autophagy adaptors (p62, NDP52) and induces autophagosome formation with autophagy machinery consisting of Unc-51-like autophagy-activating kinases (ULK) complex, phosphoinositide 3-kinase (PI3K) complex (Beclin1, VSP34, ATG14L) and the ATG16L1 complex (ATG16L1, ATG5, LC3) (21). It is becoming increasingly evident that several intracellular bacteria develop unique mechanisms to modulate autophagy, in particular to avoid autolysosomal destruction, to facilitate host colonization (15, 20). In the case of rickettsiae, the role of autophagy in regulating host colonization remains inconclusive (22 – 25). For instance, *Rickettsia australis*, a virulent member of the transitional group (TRG), benefited from ATG5-dependent autophagy and suppression of pro-inflammatory cytokine responses to colonize host cells (22, 23). In contrast, reports on *Rickettsia parkeri*, a mild-virulent member of the spotted fever group (SFG), demonstrated that its surface protein OmpB is critical for protecting against autophagic recognition, and further showed that evasion of autophagy was critical for invasion of bone marrow-derived macrophages (BMDMΦ) and wild-type (WT) mice (24, 25). Intriguingly, we reported that pathogenic members of SFG and typhus group (TG) rickettsiae secrete effectors to promote host colonization by modulating endoplasmic reticulum structures or by hijacking the autophagic defense pathway, respectively (26 – 31). In fact, our findings on the flea-transmitted *R. typhi*, a pathogenic member of the TG, showed that this intracytoplasmic pathogen is ubiquitinated upon host entry and escapes autolysosomal fusion to establish an intracytosolic niche in non-phagocytic cells (30). Thus, in agreement with reports on *R. australis* (22, 23), these findings suggest a mechanism by which highly pathogenic *Rickettsia* spp., including members of TG, TRG, and possibly other SFG rickettsiae, activate autophagy, but subsequently evade autolysosomal destruction, to promote host colonization. These unexpected findings on how SFG, TRG, and TG *Rickettsia* differentially promote their host dissemination prompted us to test the hypothesis that survival of both pathogenic *R. typhi* and *R. rickettsii* (SS), but not the non-pathogenic *Rickettsia montanensis*, involves evasion of autophagosomal maturation and reduction of microbicidal pro-inflammatory interleukin-1 (IL-1) cytokine responses to establish a replication niche in phagocytic host immune defense cells, like macrophages.

## RESULTS

### Pathogenic rickettsiae are ubiquitinated and induce autophagy upon entry into macrophages

**Fig 1:**
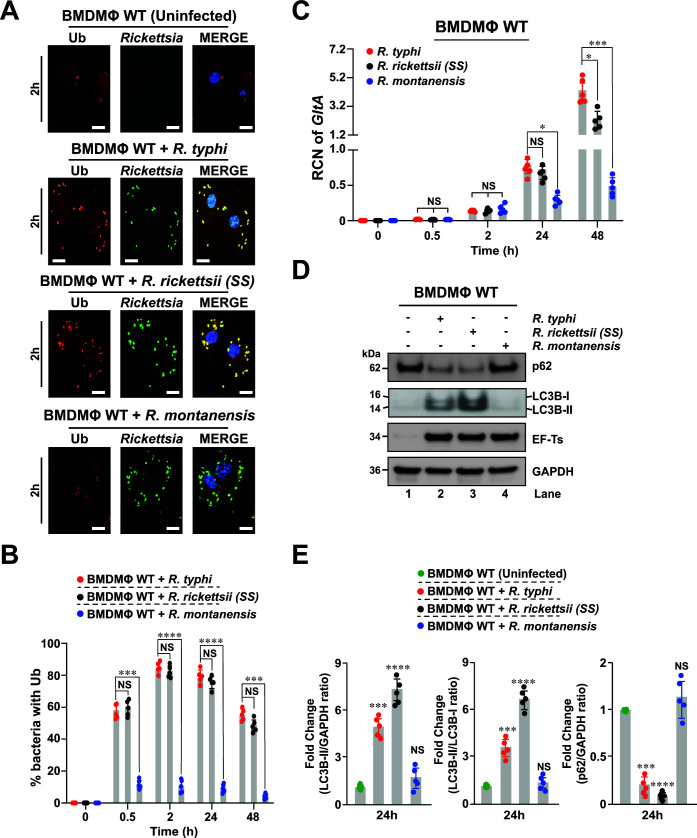
*R. rickettsii* (SS) and *R. typhi*, but not *R. montanensis*, are ubiquitinated and induced autophagy in macrophages. (**A**) BMDMΦ from WT mice were infected with *R. montanensis*, *R. rickettsii* (SS), or *R. typhi* at amultiplicity of infection (MOI) of 20 (for 0.5 and 2 h) and 5 (for 24 and 48 h). Samples were fixed with 4% paraformaldehyde and *Rickettsia* spp. were detected using specific Alexa Fluor 488-conjugated *Rickettsia* (SFG or TG) antibodies, while Ub status was assessed using Alexa Fluor 594-conjuagted anti-Ub antibody. Images represent *Rickettsia*-infected macrophages after 2 h post-infection. DNA was stained using 4´,6-diamidino-2-phenylindole (blue). Colocalization between *Rickettsia* and Ub was analyzed using Coloc 2 plugin Fiji software. Bars in panel A, 10 µm. (**B**) Graph shows the percentage of Ub positive stained *Rickettsia* at indicated time points (0.5, 2, 24, and 48 h) of infection. Approximately 100–200 bacteria were counted per strain and time point. (**C**) Bacterial burdens in *Rickettsia*-infected BMDMΦ were evaluated 0.5, 2, 24, and 48 h post-infection by *GltA* reverse transcription quantitative real-time PCR (RT-qPCR). Relative copy number (RCN) of *GltA* expression was normalized by the expression of the *GAPDH*. (**D**) *Rickettsia*-infected WT BMDMΦ, as described in panel A, were lysed and samples were immunoblotted with anti-p62, anti-LC3B, anti-elongation factor Ts (EF-Ts), and anti-GAPDH antibodies. Immunoblot data are a representative of three independent experiments. (**E**) Densitometry analysis from samples shown in panel D was performed using Fiji software and data represent the fold change between the ratios of LC3B-II/GAPDH, LC3B-II/LC3B-I, or p62/GAPDH. Error bars (B, C, E) represent means ± standard error of the mean (SEM) from five independent experiments; NS, non-significant; *P ≤ 0.05; ***P ≤ 0.005; ****P ≤ 0.001.

Preceding findings suggest that intracellular pathogens, like rickettsiae, not only encounter inflammasome-dependent defense mechanisms but are also confronted by another cytosolic defense pathway, autophagy (22 – 25, 30, 32 – 35). Both responses not only are key to mount the appropriate host defense responses, but are also functionally interconnected (15, 16). In one of our preceding reports, we showed that *R. typhi* is ubiquitinated upon host entry and induces autophagy, but escapes autophagosomal maturation for intracellular colonization in non-phagocytic cells (30). More recently, we demonstrated that unlike *R. montanensis* (a non-pathogenic SFG member), *R. rickettsii* (SS) and *R. typhi*, two highly pathogenic *Rickettsia* spp., preferentially targeted the non-canonical inflammasome-IL-1α signaling axis in macrophages to support their replication (32). Given these reports by others and our recent findings, we first evaluated the ubiquitination status of *R. typhi*, *R. rickettsii* (SS), or *R. montanensis* during invasion of bone marrow-derived macrophages isolated from C57BL/6J WT mice. Similar to infection studies using *R. australis* (23), we observed that both *R. rickettsii* (SS) and *R. typhi* spp. were ubiquitinated during the course of invasion of WT BMDMΦ (Fig. 1A and B). In contrast, but similar to *R. parkeri* infections, *R. montanensis* was not ubiquitinated in WT BMDMΦ (Fig. 1A and B). These data suggest that the life cycle of *R. montanensis* is restricted by phagolysosomal fusion allowing for only a small number of bacteria to escape into cytosol of macrophages and support the findings from another laboratory using a human monocytic Tohoku Hospital Pediatrics-1 (THP-1) cell line (36, 37). To test that hypothesis, we evaluated the bacterial burdens of *R. typhi*, *R. rickettsii* (SS), or *R. montanensis* during invasion of WT BMDMΦ and demonstrated that, unlike *R. montanensis*, both *R. rickettsii* (SS) and *R. typhi* replicated in macrophages (Fig. 1C), which is in agreement with our previous published work (32). Furthermore, we evaluated the status of autophagy markers p62 (also known as sequestosome-1 [SQSTM1]) and autophagic vesicle formation marker LC3B (38) during infection of WT BMDMΦ by Western blot analyses. Our data revealed that, unlike *R. montanensis*, infection with *R. rickettsii* (SS) and *R. typhi* induced autophagy, as evidenced by an enhanced induction of LC3B and the simultaneous down-regulation of p62 expression (Fig. 1D and E). Given these findings, we performed similar infection studies in the presence of bafilomycin A1 (Baf A1), a late-stage autophagy inhibitor, and found that treatment with Baf A1 resulted in a further increase in LC3B levels providing additional evidence for a direct modulation of autophagy during the invasion of pathogenic rickettsiae (Fig. S1).

### Pathogenic rickettsiae avoid autolysosomal destruction to establish a replicative niche in macrophages

**Fig 2:**
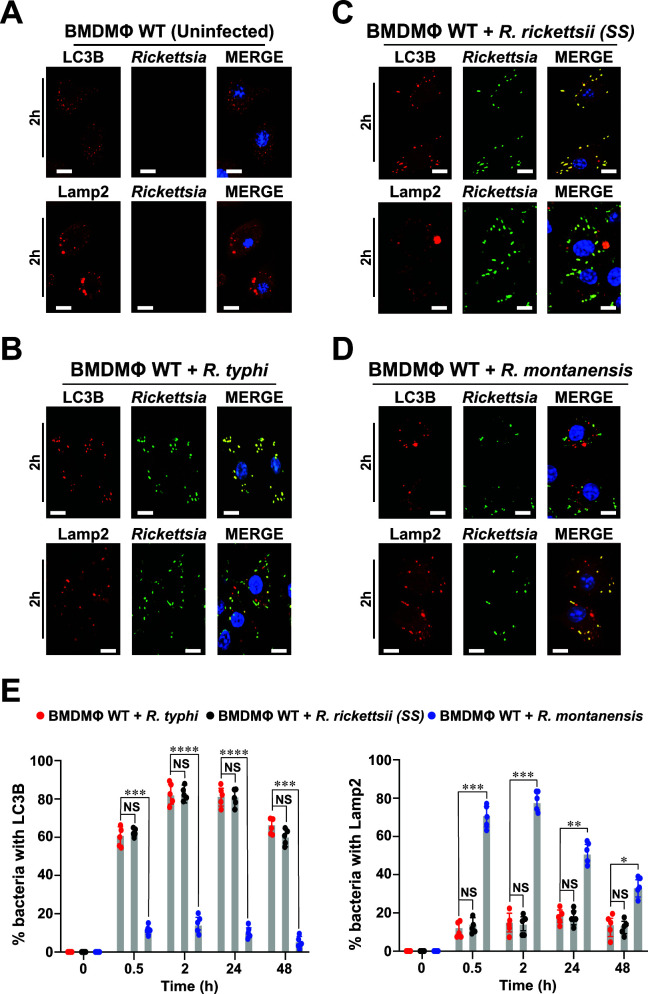
*R. rickettsii* (SS) and *R. typhi* evade autolysosomal destruction to establish a replication niche in macrophages. (**A–D**) BMDMΦ from WT mice were infected with *R. typhi*, *R. rickettsii* (SS), or *R. montanensis* at a multiplicity of infection (MOI) of 20 (for 0.5 and 2 h) and 5 (for 24 and 48 h). Cells were fixed with 4% paraformaldehyde and *Rickettsia* spp. were detected using the SFG or TG *Rickettsia*-specific antibodies conjugated with Alexa Fluor 488, while LC3B and Lamp2 expressions were assessed using Alexa Fluor 594-conjuagted anti-LC3B or anti-Lamp2 antibodies, respectively. Images represent *Rickettsia*-infected macrophages after 2 h post-infection. DNA was stained using 4´,6-diamidino-2-phenylindole (blue). Colocalization between *Rickettsia* and LC3B or Lamp2 was analyzed using Coloc 2 plugin Fiji software. Bars in panels A–D, 10 µm. (**E**) Graphs show the percentage of LC3B or Lamp2 positive stained *Rickettsia* at indicated time points (0.5, 2, 24, and 48 h) of infection. Approximately 100–200 bacteria were counted per strain and time point. Error bars (**E**) represent means ± standard error of the mean (SEM) from five independent experiments; NS, non-significant; *P ≤ 0.05; **P ≤ 0.01; ***P ≤ 0.005; ****P ≤ 0.001.

Autophagy is an intracellular process that delivers autophagosomes to the lysosomes for degradation and is considered as one of the host defense pathways to combat bacterial infection, including other cellular functions (19, 20, 39). Given our above presented data that pathogenic, but not non-pathogenic, *Rickettsia* spp. are ubiquitinated upon host cell entry (Fig. 1A and B), we next evaluated the status of autophagy marker, LC3B, and lysosomal marker, Lamp2, during infection of WT BMDMΦ by immunofluorescence assay (IFA). We observed that, unlike *R. montanensis*, *R. typhi* and *R. rickettsii* (SS) colocalized with autophagy marker, LC3B, over the course of infection (Fig. 2A through D). As we previously demonstrated that *R. typhi* was able to avoid autolysosomal destruction in non-phagocytic cells (30), we next assessed the colocalization pattern of all three *Rickettsia* spp. with Lamp2. We observed that, unlike *R. montanensis*, both *R. typhi* and *R. rickettsii* (SS) spp. did not colocalize with Lamp2 in WT BMDMΦ (Fig. 2A through E), suggesting that both pathogenic *Rickettsia* spp. induce autophagy and escape autolysosomal destruction to facilitate their intracytosolic survival.

### Intracytosolic survival of pathogenic rickettsiae requires induction of autophagy and reduction of microbicidal pro-inflammatory IL-1 cytokine responses

**Fig 3:**
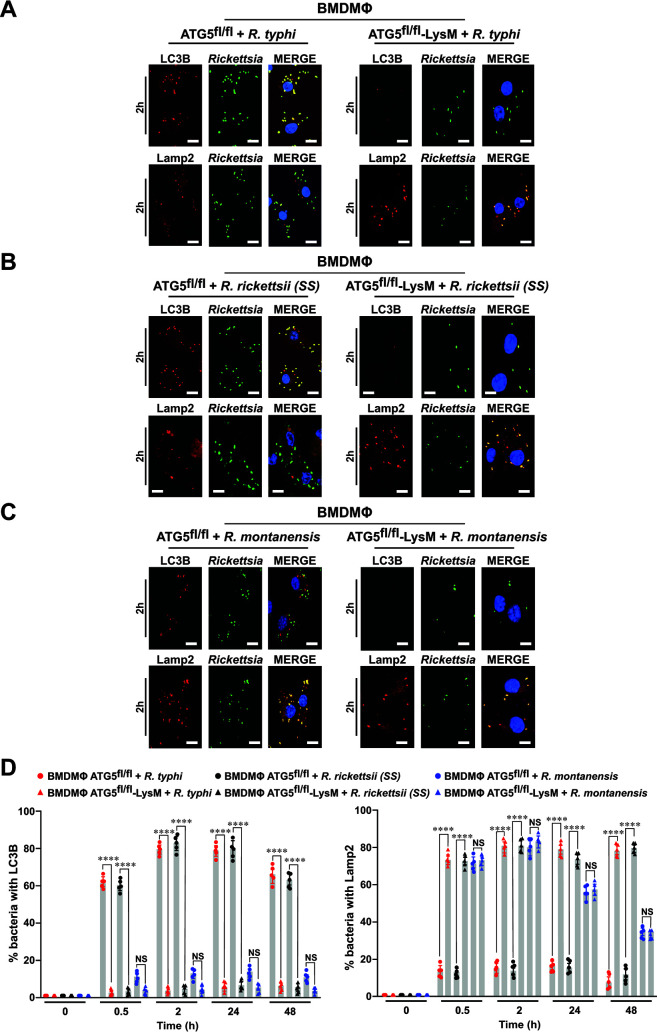
Intracytosolic survival of *R. rickettsii* (SS) and *R. typhi* involves autophagy and evasion of autolysosomal destruction. (**A–C**) BMDMΦ from ATG5^fl/fl^ (WT) or ATG5^fl/fl^-LysM-Cre (knockout [KO]) mice were infected with *R. typhi*, *R. rickettsii* (SS), or *R. montanensis* at a multiplicity of infection (MOI) of 20 (for 0.5 and 2 h) and 5 (for 24 and 48 h). Cells were fixed with 4% paraformaldehyde and *Rickettsia* were detected using the SFG or TG *Rickettsia*-specific antibodies conjugated with Alexa Fluor 488, while LC3B and Lamp2 expressions were assessed using Alexa Fluor 594-conjuagted anti-LC3B or anti-Lamp2 antibodies, respectively. Images represent *Rickettsia*-infected macrophages after 2 h post-infection. DNA was stained using 4´,6-diamidino-2-phenylindole (blue). Colocalization between *Rickettsia* and LC3B or Lamp2 was analyzed using Coloc 2 plugin Fiji software. Bars in panels A–C, 10 µm. (**D**) Graphs show the percentage of LC3B or Lamp2 positive stained *Rickettsia* at indicated time points (0.5, 2, 24, and 48 h) of infection. Approximately 100–200 bacteria were counted per strain and time point. Error bars (**D**) represent means ± standard error of the mean (SEM) from five independent experiments; NS, non-significant; ****P ≤ 0.001.

Based on recent findings from other laboratories and ours (22 – 25, 30, 32 – 35, 40), it has become evident that *Rickettsia* spp. exhibit variable degree of pathogenicity, indicating species-specific strategies to respond to mechanism of host defense surveillance, including autophagy and inflammasome responses. To address the role of autophagy in modulating the intracellular survival of *Rickettsia* spp., we utilized BMDMΦ from mice genetically lacking the ATG5 gene (ATG5^fl/fl^-LysM-Cre) in myeloid cells, a well-accepted mutant model defective in the autophagy pathway (41). Next, we determined the colocalization status of *R. montanensis*, *R. typhi*, and *R. rickettsii* (SS) with LC3B during infection of ATG5^fl/fl^ or ATG5^fl/fl^-LysM-Cre BMDMΦ by IFA. We found that, unlike *R. montanensis*, both *R. typhi* and *R. rickettsii* (SS) strains colocalized with LC3B in ATG5^fl/fl^ BMDMΦ, and not in ATG5^fl/fl^-LysM-Cre BMDMΦ (Fig. 3A through D; Fig. S2). Moreover, we observed that, unlike *R. typhi* and *R. rickettsii* (SS), *R. montanensis* colocalized with Lamp2 in ATG5^fl/fl^ BMDMΦ (Fig. 3A through D; Fig. S2). However, all three *Rickettsia* strains colocalized with Lamp2 in ATG5^fl/fl^-LysM-Cre BMDMΦ (Fig. 3A through D; Fig. S2). As it has been reported that ATG5 can modulate other host events independently of autophagy (42), we employed BMDMΦ from mice genetically deprived in the Beclin1 gene function (Beclin1^fl/fl^-LysM-Cre) in myeloid cells, an alternative mutant model defective in the autophagy pathway (41). Accordingly, we determined the colocalization status of *R. montanensis*, *R. typhi*, and *R. rickettsii* (SS) with LC3B and Lamp2 during infection of Beclin1^fl/fl^-LysM-Cre or Beclin1^fl/fl^ (corresponding WT control) BMDMΦ by IFA. In line with our infection studies using ATG5^fl/fl^ and ATG5^fl/fl^-LysM-Cre BMDMΦ, we found that, unlike *R. montanensis*, both *R. typhi* and *R. rickettsii* (SS) strains colocalized with LC3B in Beclin1^fl/fl^ BMDMΦ, and not in Beclin1^fl/fl^-LysM-Cre BMDMΦ (Fig. S3). Furthermore, we observed that, unlike *R. typhi* and *R. rickettsii* (SS), *R. montanensis* colocalized with Lamp2 in Beclin1^fl/fl^ BMDMΦ (Fig. S3), while all three *Rickettsia* spp. colocalized with Lamp2 in Beclin1^fl/fl^-LysM-Cre BMDMΦ (Fig. S3), suggesting that the intracytosolic survival of both pathogenic *R. typhi* and *R. rickettsii* (SS) strains in macrophages requires the activation of autophagy in an ATG5-/Beclin1-dependent manner and escape from autolysosomal destruction.

**Fig 4:**
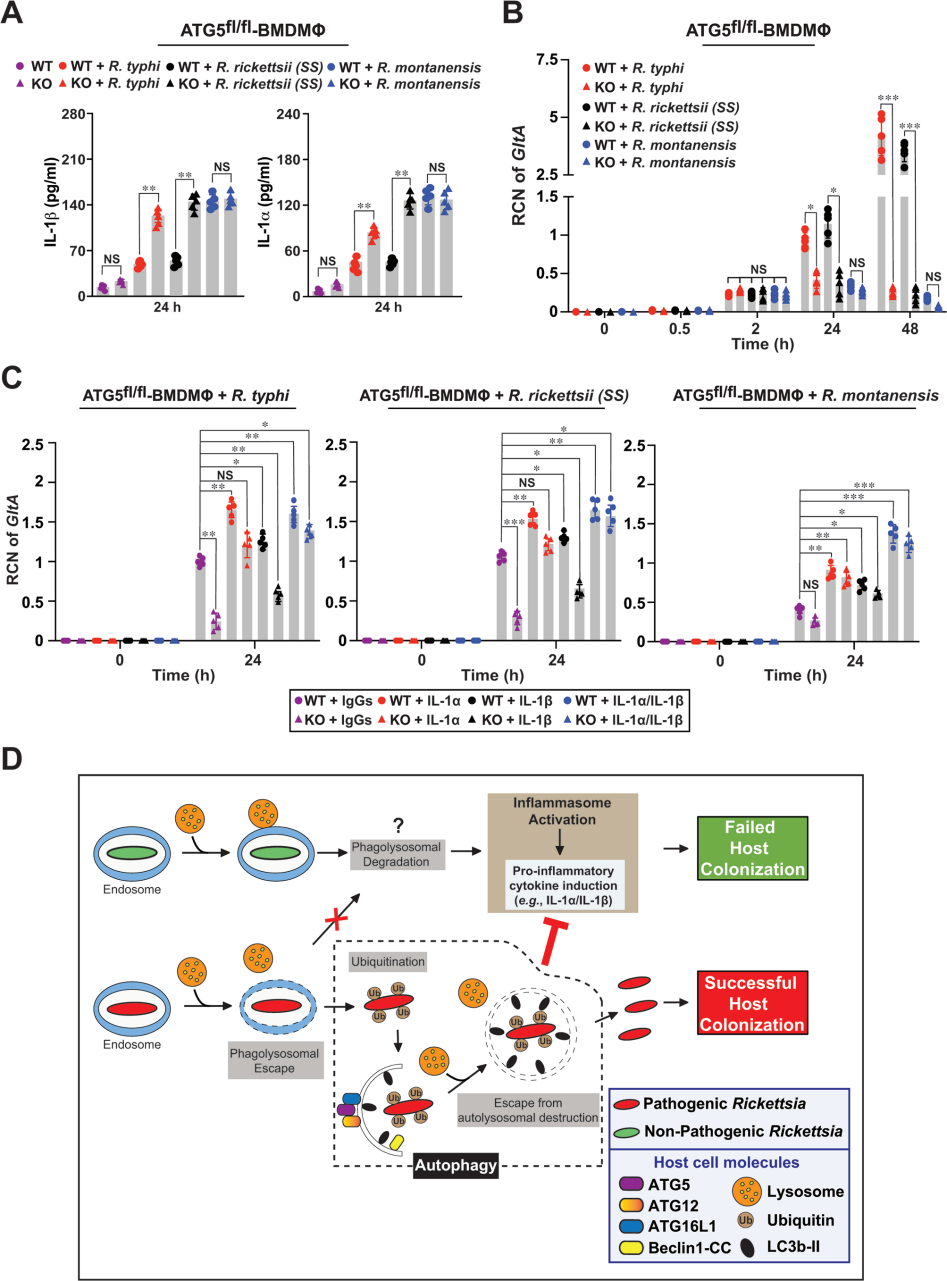
*R. rickettsii* (SS) and *R. typhi*, but not *R. montanensis*, utilize autophagy induction and subsequent inhibition of microbicidal pro-inflammatory IL-1 responses to establish a replication niche. (**A**) Culture supernatants of uninfected and *R. typhi*-, *R. rickettsii* (SS)*-*, or *R. montanensis*-infected ATG5^fl/fl^ (WT) and ATG5^fl/fl^-LysM-Cre [Knockout (KO)] BMDMΦ were analyzed 24 h post-infection to determine the level of IL-1β and IL-1α cytokines using Legendplex kits (BioLegend) followed by flow cytometry. (**B**) Bacterial burdens in infected BMDMΦ were evaluated 0.5, 2, 24, and 48 h post-infection by *GltA* reverse transcription quantitative real-time PCR (RT-qPCR). Expression of the host housekeeping gene *GAPDH* was used for normalization. (**C**) Bacterial burdens in *Rickettsia*-infected ATG5^fl/fl^ or ATG5^fl/fl^-LysM-Cre treated with anti-IL-1α, IL-1β, both anti-IL-1α and anti-IL-1β antibodies, or IgG isotype controls. Samples were evaluated at 24 h post-infection by *GltA* RT-qPCR. Expression of the host housekeeping gene *GAPDH* was used for normalization. Error bars (**A–C**) represent means ± standard error of the mean (SEM) from five independent experiments; NS, non-significant; *P ≤ 0.05; **P ≤ 0.01; ***P ≤ 0.005. (**D**) Proposed working model on how pathogenic *Rickettsia* spp. initiate autophagy, evade autolysosomal destruction, and suppress IL-1 cytokine responses to establish an intracytosolic replication niche in macrophages. Of note, a sizable amount of non-pathogenic *Rickettsia* is likely destroyed by phagolysosomal fusion, while a subpopulation may escape lysosomal fusion, ultimately resulting in the induction of IL-1 responses.

Our recent findings identified a previously unappreciated mechanism by which *R. typhi* and *R. rickettsii* (SS), unlike *R. montanensis*, benefited from a reduced IL-1 cytokine response, specifically IL-1α, to support their replication within the host (32). Intriguingly, *R. australis* benefited from ATG5-dependent autophagy induction and reduction of pro-inflammatory cytokine responses (22, 23); however, a successful host colonization of *R. parkeri* involved the evasion of autophagy and inflammasome-mediated host cell death (24, 25), leaving the precise mechanism inconclusive. To gain further mechanistic insight, we first evaluated the IL-1α and IL-1β cytokine responses in cultured supernatants of *R. montanensis*-, *R. typhi*-, and *R. rickettsii* (SS)-infected ATG5^fl/fl^ BMDMΦ, as well as ATG5^fl/fl^-LysM-Cre BMDMΦ. We observed that infection of ATG5^fl/fl^ BMDMΦ with *R. typhi* or *R. rickettsii* (SS) produced significantly lower levels of IL-1β or IL-1α cytokine, as compared to that of *R. montanensis* (Fig. 4A). Infection of ATG5^fl/fl^-LysM-Cre BMDMΦ resulted in an increase of IL-1β or IL-1α cytokine levels for both *R. typhi* and *R. rickettsii* (SS) (Fig. 4A). Furthermore, we observed that, unlike *R. montanensis*, both *R. typhi* and *R. rickettsii* (SS) replicated in infected ATG5^fl/fl^ BMDMΦ; however, the replication of both bacteria was significantly impaired in ATG5^fl/fl^-LysM-Cre BMDMΦ (Fig. 4B). Next, we validated our IL-1β or IL-1α cytokine and bacterial burden data in *R. montanensis-*, *R. typhi*-, or *R. rickettsii* (SS)-infected Beclin1^fl/fl^ BMDMΦ and Beclin1^fl/fl^-LysM-Cre BMDMΦ. Our assays showed that infection of Beclin1^fl/fl^ BMDMΦ with *R. typhi* or *R. rickettsii* (SS) produced lower IL-1β or IL-1α cytokine levels, as compared to that of *R. montanensis* (Fig. S4A). Infection of Beclin1^fl/fl^-LysM-Cre BMDMΦ with *Rickettsia* spp. resulted in an increase of IL-1β or IL-1α cytokine levels for both *R. typhi* and *R. rickettsii* (SS) (Fig. S4A). Furthermore, we found that, unlike *R. montanensis*, both *R. typhi* and *R. rickettsii* (SS) replicated in infected Beclin1^fl/fl^ BMDMΦ; however, the replication of both bacteria was impaired in Beclin1^fl/fl^-LysM-Cre BMDMΦ (Fig. S4B). Given these data, we sought to determine the biological importance of autophagy induction and IL-1β or IL-1α cytokine responses in modulating the survival of all three *Rickettsia* spp. in macrophages. In this effort, we neutralized the activity of IL-1β or IL-1α cytokines using anti-IL-1β or anti-IL-1α antibodies and assessed the effect on the bacterial burdens for *R. montanensis*-, *R. typhi*-, and *R. rickettsii* (SS)-infected ATG5^fl/fl^ BMDMΦ, as well as ATG5^fl/fl^-LysM-Cre BMDMΦ. Neutralization of IL-1α and IL-1β (with a lower effectiveness) resulted in an increase in bacterial loads of *R. typhi*-, *R. rickettsii* (SS)-, and *R. montanensis*-infected ATG5^fl/fl^ BMDMΦ (Fig. 4C). Importantly, antibody neutralization of IL-1α and IL-1β (with a lower effectiveness), increased the bacterial burdens of *R. typhi*-, and *R. rickettsii* (SS)-infected ATG5^fl/fl^-LysM-Cre BMDMΦ, reaching levels observed in infected ATG5^fl/fl^ BMDMΦ (Fig. 4C). Of note, concurrent anti-IL-1α and anti-IL-1β antibody treatments in *R. typhi*- and *R. rickettsii* (SS)-infected ATG5^fl/fl^ BMDMΦ or ATG5^fl/fl^-LysM-Cre BMDMΦ did not result in a synergistic effect (Fig. 4C). Neutralization of either IL-1α or IL-1β (with a lower effectiveness) cytokine resulted in an increase in the bacterial loads of *R. montanensis*-infected ATG5^fl/fl^ BMDMΦ or ATG5^fl/fl^-LysM-Cre BMDMΦ (Fig. 4C), while concurrent treatment with both antibodies resulted in synergistic increase in bacteria loads (Fig. 4C). The efficiency of antibody-mediated blocking was validated by comparing the levels of IL-1β and IL-1α cytokines in the supernatants of IgG isotype control, IL-1β and IL-1α antibody-treated *Rickettsia*-infected ATG5^fl/fl^ BMDMΦ, as well as ATG5^fl/fl^-LysM-Cre BMDMΦ (Fig. S5).

Collectively, the data presented here are in agreement with prior reports from our laboratory and others (22, 23, 30, 32), further confirming that the intracytosolic survival of *R. typhi* and *R. rickettsii* (SS), but not *R. montanensis*, in macrophages requires the activation of autophagy in an ATG5-/Beclin1-dependent manner, escape from autolysosomal destruction, and inhibition of IL-1α and IL-1β cytokines responses (Fig. 4D).

## DISCUSSION

Various intracellular bacterial pathogens employ sophisticated mechanisms to hijack host cellular processes to facilitate their host survival. Such strategies entail reprogramming host phosphoinositide metabolism, which can facilitate uptake into host cells, modify phagosomes, undercut apoptosis, and interfere with other cellular defense mechanisms, including inflammasomes and autophagy. However, in the case of rickettsiae, the mechanisms by which these intracytosolic pathogens modulate both inflammasome and autophagy responses to facilitate their replication in endothelial cells and immune cells, like macrophages, are only now emerging (22 – 25, 30, 32 – 35, 40, 43). In fact, recent findings from others and our laboratory have provided some intriguing findings that may suggest that *Rickettsia* spp. exhibit species-specific inflammasome-mediated immune responses to establish an intracellular niche (22 – 24, 32, 34). However, the precise role of autophagy in regulating host colonization by *Rickettsia* spp. remains to be determined (22 – 25). For instance, *R. australis* benefited from autophagy induction and reduction of pro-inflammatory cytokine responses (22, 23), while *R. parkeri* evaded autophagic responses to colonize the host (24, 25). Our recent reporting on *R. typhi* showed that ubiquitination followed by autophagy induction, and the escape from autolysosomal destruction, were crucial steps for *R. typhi* to colonize non-phagocytic cells (30). Based on these findings from others and our laboratory, we tested the hypothesis that both pathogenic *R. typhi* and *R. rickettsii* (SS), but not the non-pathogenic *R*. montanensis, induce autophagy, evade autolysosomal destruction, and reduce microbicidal pro-inflammatory IL-1 responses to establish an intracytosolic replication niche in host immune defense cells, like macrophages.

Our data revealed that, using partially purified rickettsiae, *R. montanensis* is not ubiquitinated, while both *R. typhi* and *R. rickettsii* (SS) are ubiquitinated, during infection of macrophages. Ubiquitination of *R. typhi* and *R. rickettsii* (SS) is in agreement with infection studies using the Renografin-purified *R. australis* spp. (23). In contrast, no ubiquitination of partially purified *R. montanensis* was observed in our assays, which is in agreement with reports using the mild-pathogenic *R. parkeri* spp., where a MD-76R (overlaid or step gradient)-purified bacterium was used for ubiquitination studies (24, 25). Collectively, these findings by our laboratory and others suggest that variations in the intracellular behavior of *Rickettsia* spp. are likely due to species-specific strategies employed by the bacteria and not because of differences in the purification methodologies. However, the precise mechanism of these phenotypic differences on rickettsial ubiquitination requires further investigation. In addition, our data demonstrated that, unlike *R. montanensis*, both *R. typhi* and *R. rickettsii* (SS) spp. induced autophagy and evaded lysosomal destruction during macrophage invasion. Importantly, our presented data using ATG5-/Beclin1-deficient macrophages further strengthen our hypothesis that *R. typhi* and *R. rickettsii* (SS) utilize autophagy for establishing an intracytosolic replication niche in macrophages (Fig. 4D).

Our findings also raised another intriguing question as to why both *R. typhi* and *R. rickettsii* (SS) spp. seem to possess the ability to actively induce autophagy, in contrast to *R. montanensis*. Furthermore, our data suggest that both *R. typhi* and *R. rickettsii* (SS) spp. can efficiently escape from phagolysosomal destruction, become ubiquitinated, and induce autophagy. However, *R. montanensis* localized with Lamp2^+^-lysosomes and failed to evade lysosomal destruction, a phenotype previously observed in THP-1 cells (36, 37), suggesting that the phagolysosomal maturation likely contributes to the lack of ubiquitination and autophagic recognition of *R. montanensis*. Also, as *Rickettsia*-specific immunodominant outer membrane proteins (e.g., Scas) are predicted to be expressed on either pathogenic or non-pathogenic *Rickettsia* spp. (44), another factor to consider is the presence of an effector repertoire that differs depending on the rickettsial virulence. In fact, we recently reported that *R. typhi* induces autophagy upon infection of non-phagocytic cells, while subsequently avoiding autolysosomal destruction, via the function of secreted effectors, including phosphatidylinositol 3-kinase Risk1 and phospholipases Pat1 and Pat2 (30, 44). This mechanism of host invasion seems consistent with rickettsiae close relatives, such as *Anaplasma phagocytophilum* and *Ehrlichia chaffeensis* (45 – 48), or other intracellular pathogens such as *Shigella* (49, 50). So, it is conceivable that effectors (e.g., Risk1 and Pat1/2 [30, 44]), either alone or in combination with other currently yet to be identified effectors, could account for the observed phenotypic differences in host colonization among *Rickettsia* spp. (*R. montanensis*, *R. typhi,* and *R. rickettsii* [SS]). The precise mechanism and composition of the effector repertoire for each *Rickettsia* spp. are currently a matter of active research and still remain to be determined.

Our preceding report suggest that IL-1 signaling responses played a role in limiting rickettsial infection *in vivo* and *in vitro* (32). Specifically, we observed that both *R. typhi* and *R. rickettsii* (SS), but not *R. montanensis*, blocked cell death and reduced non-canonical inflammasome-dependent IL-1α responses, in order to establish an intracytosolic replication niche in macrophages. In line with these findings, we now report that the *R. montanensis*, but not *R. typhi* and *R. rickettsii* (SS), is efficiently cleared by macrophages, a mechanism supported by previously published findings with various SFG *Rickettsia* spp. using THP-1 cells (36, 37). Another intriguing observation is that the survival of both *R. typhi* and *R. rickettsii* (SS) was restricted in ATG5- or Beclin1-deficient macrophages, as evident by their localization with destructive Lamp2^+^-lysosomes and the increase in pro-inflammatory IL-1 cytokine response. These findings suggests that activation of autophagy is critical for the intracellular survival of both *R. typhi* and *R. rickettsii* (SS), likely via an autophagy-mediated proteasomal degradation of pro-inflammatory IL-1 cytokines and/or signaling components/molecules involved in their induction (17 – 19), a mechanism supporting the report on *R. australis* (23). However, the precise mechanism on how the intracellular survival of *R. typhi* and *R. rickettsii* (SS) benefits from the activation of an ATG5-/Beclin1-dependent autophagy pathway to limit pro-inflammatory IL-1 cytokine responses remains unclear and requires further investigation. Given the data presented in this manuscript, our recent report (30, 32), and published reports by others (13, 14, 17, 19, 22, 23), we now present a working model of host invasion by which both pathogenic *R. typhi* and *R. rickettsii* (SS) become ubiquitinated, induce autophagy, avoid autolysosomal destruction, and reduce microbicidal IL-1 cytokine responses to establish an intracytosolic niche in macrophages (Fig. 4D).

## MATERIALS AND METHODS

### Antibodies and reagents

Mono- and polyubiquitinylated conjugated antibody (clone: FK1) (anti-Ub) was obtained from Enzo Life Sciences. Anti-LC3B (E5Q2K) antibody was from Cell Signaling Technology. Antibodies against whole *Rickettsia* (SFG or TG) were raised in-house. Antibody against rickettsial cytoplasmic protein elongation factor Ts (EF-Ts) was obtained from Primm Biotech as previously described (30). The p62/SQSTM1 antibody was purchased from Sigma. Lamp2 (H4B4) and GAPDH (FL-335) antibodies were from Santa Cruz Biotechnology. ProLong Gold antifade mounting medium with DAPI (4´,6-diamidino-2-phenylindole), Halt protease and phosphatase inhibitor cocktail, paraformaldehyde (PFA), and Alexa 488/594-conjugated secondary antibodies were purchased from Thermo Fisher Scientific.

### Bacterial strains, cell culture, and infection

Vero76 cells (African green monkey kidney, American Type Culture Collection [ATCC], RL-1587) were maintained in minimal Dulbecco’s modified Eagle’s medium (DMEM) supplemented with 10% heat-inactivated fetal bovine serum (FBS) at 37°C with 5% CO_2_. *R. montanensis* (M5/6) and *R. rickettsii* (SS) strains were obtained from Dr. Ted Hackstadt (Rocky Mountain Laboratories, NIH, MT, USA), while the *R. typhi* strain (Wilmington) was obtained from CDC. All *Rickettsia* strains were propagated in Vero76 cells grown in DMEM supplemented with 5% FBS at 34°C with 5% CO_2_. All *Rickettsia* strains were partially purified as described previously (27, 51). Partially purified *R. montanensis*, *R. rickettsii* (SS), and *R. typhi* were used for infection of BMDMΦ at 34°C. At early stages of infection (before the doubling time [8 to 10 h]) of rickettsiae), a higher multiplicity of infection (MOI) of 20 (for 0.5 and 2 h post-infection [hpi]) was used to ensure the presence of sufficient number of bacteria, as compared to MOI of 5 at later time points (24 and 48 hpi), to determine the biological functions of the bacteria during host infection (26 – 28, 30, 32).

### Differentiation of bone marrow-derived macrophages

Femurs and tibias from 8- to 10-week-old female C57BL/6J WT mice were obtained as described previously (32, 52). Femurs and tibias from female C57BL/6J conditional gene knockout mice, Atg5^fl/fl^-LysM-Cre and Beclin-1^fl/fl^-LysM-Cre mice, in which the Atg5 or Beclin1 gene was deleted from myeloid cells (mainly monocyte/macrophages), as well as their corresponding WT littermates (ATG5^fl/fl^ or Beclin1^fl/fl^), were provided by Dr. Christina Stallings (Washington University School of Medicine, MO, USA) (41). Isolated bone marrow cells were differentiated in RPMI 1640 medium supplemented with 10% FBS and 30% L929-conditioned medium (a source of macrophage colony stimulating factor) and cultured for 7 days as described previously (32, 52).

### Measurement of cytokines and chemokines

IL-1 cytokine concentrations in supernatants from cultured BMDMΦ were assessed using the Legendplex mouse inflammation kit (BioLegend) following the manufacturer’s instructions as described previously (32, 52).

### RNA isolation and quantitative real-time PCR

To determine viable bacterial number during the course of host infection, we performed Real time-quantitative polymerase chain reaction (RT-qPCR) assay on isolated RNA (26, 53, 54). In this effort, BMDMΦ samples were collected at 0.5, 2, 24, and 48 h post-infection. RNA was extracted from 1 × 10^6^ BMDMΦ using the Quick-RNA miniprep kit (Zymo Research). The iScript Reverse Transcription Supermix kit (Bio-Rad; 1708841) was used to synthesize cDNAs from 200 ng of RNA according to the manufacturer’s instructions. Quantitative real-time PCR was performed using SYBR Green (Thermo Fisher Scientific), 2 µL cDNA, and 1 µM each of the following oligonucleotides for rickettsial (housekeeping) citrate synthase gene (*GltA*), and host (housekeeping) *GAPDH* gene. Relative copy number (RCN) of *GltA* expression was normalized by the expression of the *GAPDH* and calculated with the equation *RCN* = *E* ^−Δ*Ct*^, where *E* = efficiency of PCR and *Ct* = Ct *target* − Ct *GAPDH*. Melting curve analyses were performed at the end of each run to ensure that only one product was amplified as described previously (32, 55).

### Immunofluorescence

Eight-well chamber slides were seeded with BMDMΦ (30–50 × 10^4^ cells per well) and infected with partially purified pathogenic and non-pathogenic *Rickettsia* spp. (MOI = 20 [0.5 and 2 h] and MOI = 5 [24 and 48 h]) as described previously (26 – 28, 30, 32). Briefly, partially purified rickettsiae were added to BMDMΦ and incubated for various lengths of time at 34°C with 5% CO_2_. Following incubation, cells were washed three times with 1× phosphate-buffered saline (PBS) and fixed with 4% PFA for 20 min at room temperature. Cells were than permeabilized in blocking buffer (0.3% saponin and 0.5% normal goat serum in 1× PBS) for 30 min and incubated for 1 h with the following primary antibodies diluted in antibody dilution buffer (0.3% saponin in 1× PBS): anti-*Rickettsia* (1:100 or 1:500), anti-Ub (1:100), anti-LC3B (1:100), and anti-Lamp2 (1:100). Cells were then washed with 1× PBS and incubated for 1 h with anti-Alexa Fluor 488 or anti-Alexa Fluor 594 secondary antibodies diluted 1:1,500 in antibody dilution buffer. Next, cells were washed with 1× PBS and mounted with ProLong Gold antifade mounting medium containing DAPI. Images were acquired using the Nikon W-1 spinning disk confocal microscope (University of Maryland Baltimore, Confocal Core Facility) and degree of colocalization (yellow^+^-stained bacteria) between *Rickettsia* and ubiquitin; LC3B or Lamp2 was analyzed using Fiji software as described previously (30). Quantification of the mean percentage of cellular marker positive bacteria was calculated from approximately 100 host cells for each experiment, while each experiment was repeated five times. The percentage of internalized bacteria (approximately 100–200 bacteria were counted per strain and time point) was calculated by dividing the number of extracellular bacteria by the total number of bacteria, multiplying by 100, and then subtracting this number from 100% to get the percentage of intracellular bacteria.

### Extract preparation and Western blot analysis

*Rickettsia*-infected BMDMΦ cells were lysed for 2 h at 4°C in ice-cold lysis buffer (50  mM HEPES [pH 7.4], 137  mM NaCl, 10% glycerol, 1  mM EDTA, 0.5% NP-40, and supplemented with protease and phosphatase inhibitory cocktails) as described previously (30, 32). Equal amounts of protein were loaded for SDS-PAGE and membranes were probed with anti-p62, anti-LC3B, anti-GAPDH, and anti-EF-Ts antibodies, followed by enhanced chemiluminescence with secondary antibodies conjugated to horseradish peroxidase.

### Neutralization of IL-1α and IL-1β *in vitro*

For *in vitro* neutralization of IL-1α and IL-1β, *R. typhi-*, *R. rickettsii* (SS)-, or *R. montanensis*-infected BMDMΦ from C57BL/6J ATG5^fl/fl^ or ATG5^fl/fl^-LysM-Cre mice were treated with 100 nM anti-IL-1α (clone ALF-161; BioXCell), anti-IL-1β (clone B122; BioXCell), or IgG isotype control (Armenian hamster IgGs; BioXCell) antibodies for 24 h, and bacterial burden was assessed by quantitative real-time PCR.

### Statistical analysis

The statistical significance was assessed using analysis of variance with Bonferroni’s procedure and Student’s *t*-test (GraphPad Prism Software, version 8). Data are presented as mean ± standard error of the mean (SEM), unless stated otherwise. Alpha level was set to 0.05.

## SUPPLEMENTAL MATERIAL

Fig. S1 spectrum.02791-23-s0001.eps Treatment with a late-stage autophagy inhibitor, bafilomycin A1, results in an increase in LC3B level during rickettsiae invasion.

Fig. S2 spectrum.02791-23-s0002.eps LC3B and Lamp2 staining in uninfected ATG5^fl/fl^ and ATG5^fl/fl^-LysM-Cre macrophages.

Fig. S3 spectrum.02791-23-s0003.eps Beclin1-dependent autophagy is involved in the intracytosolic survival of both *R. rickettsii* (SS) and *R. typhi* species in macrophages.

Fig. S4 spectrum.02791-23-s0004.eps Assessment of IL-1α and IL-1β cytokine responses and intracellular survival of *R. rickettsii* (SS), *R. typhi*, and *R. montanensis* species in Beclin1^fl/fl^ and Beclin1^fl/fl^-LysM-Cre macrophages.

Fig. S5 spectrum.02791-23-s0005.eps Assessment of IL-1α and IL-1β cytokine responses in *Rickettsia*-infected ATG5^fl/fl^ and ATG5^fl/fl^-LysM-Cre macrophages treated with IL-1α, IL-1β, or IgG-isotype control antibodies.

ASM does not own the copyrights to Supplemental Material that may be linked to, or accessed through, an article. The authors have granted ASM a non-exclusive, world-wide license to publish the Supplemental Material files. Please contact the corresponding author directly for reuse.

## References

[1] Personnic N , Bärlocher K , Finsel I , Hilbi H . 2016. Subversion of retrograde trafficking by translocated pathogen effectors. Trends Microbiol 24:450–462. doi:10.1016/j.tim.2016.02.003 26924068

[2] Ray K , Marteyn B , Sansonetti PJ , Tang CM . 2009. Life on the inside: the intracellular lifestyle of cytosolic bacteria. Nat Rev Microbiol 7:333–340. doi:10.1038/nrmicro2112 19369949

[3] Gillespie JJ , Kaur SJ , Rahman MS , Rennoll-Bankert K , Sears KT , Beier-Sexton M , Azad AF . 2015. Secretome of obligate intracellular Rickettsia. FEMS Microbiol Rev 39:47–80. doi:10.1111/1574-6976.12084 25168200 PMC4344940

[4] Sahni A , Fang R , Sahni SK , Walker DH . 2019. Pathogenesis of rickettsial diseases: pathogenic and immune mechanisms of an endotheliotropic infection. Annu Rev Pathol 14:127–152. doi:10.1146/annurev-pathmechdis-012418-012800 30148688 PMC6505701

[5] Sanchez-Vicente S , Tagliafierro T , Coleman JL , Benach JL , Tokarz R . 2019. Polymicrobial nature of tick-borne diseases. mBio 10:e02055-19. doi:10.1128/mBio.02055-19 31506314 PMC6737246

[6] Bermúdez CSE , Troyo A . 2018. A review of the genus Rickettsia in Central America. Res Rep Trop Med 9:103–112. doi:10.2147/RRTM.S160951 30050361 PMC6047601

[7] Levin ML , Killmaster L , Zemtsova G , Grant D , Mumcuoglu KY , Eremeeva ME , Dasch GA . 2009. Incongruent effects of two isolates of Rickettsia conorii on the survival of Rhipicephalus sanguineus ticks. Exp Appl Acarol 49:347–359. doi:10.1007/s10493-009-9268-9 19421877

[8] Drexler N , Miller M , Gerding J , Todd S , Adams L , Dahlgren FS , Bryant N , Weis E , Herrick K , Francies J , Komatsu K , Piontkowski S , Velascosoltero J , Shelhamer T , Hamilton B , Eribes C , Brock A , Sneezy P , Goseyun C , Bendle H , Hovet R , Williams V , Massung R , McQuiston JH . 2014. Community-based control of the brown dog tick in a region with high rates of rocky mountain spotted fever, 2012-2013. PLoS One 9:e112368. doi:10.1371/journal.pone.0112368 25479289 PMC4257530

[9] Billeter SA , Metzger ME . 2017. Limited evidence for Rickettsia felis as a cause of zoonotic flea-borne rickettsiosis in Southern California. J Med Entomol 54:4–7. doi:10.1093/jme/tjw179 28082625

[10] Blanton LS , Idowu BM , Tatsch TN , Henderson JM , Bouyer DH , Walker DH . 2016. Opossums and cat fleas: new insights in the ecology of murine typhus in Galveston, Texas. Am J Trop Med Hyg 95:457–461. doi:10.4269/ajtmh.16-0197 27273642 PMC4973200

[11] Hackstadt T . 1996. The biology of rickettsiae. Infect Agents Dis 5:127–143. 8805076

[12] Hackstadt T . 1998. The diverse habitats of obligate intracellular parasites. Curr Opin Microbiol 1:82–87. doi:10.1016/s1369-5274(98)80146-x 10066459

[13] Jounai N , Kobiyama K , Shiina M , Ogata K , Ishii KJ , Takeshita F . 2011. NLRP4 negatively regulates autophagic processes through an association with Beclin1. J Immunol 186:1646–1655. doi:10.4049/jimmunol.1001654 21209283

[14] Yu J , Nagasu H , Murakami T , Hoang H , Broderick L , Hoffman HM , Horng T . 2014. Inflammasome activation leads to Caspase-1-dependent mitochondrial damage and block of mitophagy. Proc Natl Acad Sci U S A 111:15514–15519. doi:10.1073/pnas.1414859111 25313054 PMC4217429

[15] Seveau S , Turner J , Gavrilin MA , Torrelles JB , Hall-Stoodley L , Yount JS , Amer AO . 2018. Checks and balances between autophagy and inflammasomes during infection. J Mol Biol 430:174–192. doi:10.1016/j.jmb.2017.11.006 29162504 PMC5766433

[16] Krakauer T . 2019. Inflammasomes, autophagy, and cell death: the trinity of innate host defense against intracellular bacteria. Mediators Inflamm 2019:2471215. doi:10.1155/2019/2471215 30728749 PMC6341260

[17] Sun Q , Fan J , Billiar TR , Scott MJ . 2017. Inflammasome and autophagy regulation: a two-way street. Mol Med 23:188–195. doi:10.2119/molmed.2017.00077 28741645 PMC5588408

[18] Pang Y , Wu L , Tang C , Wang H , Wei Y . 2022. Autophagy-inflammation interplay during infection: balancing pathogen clearance and host inflammation. Front Pharmacol 13:832750. doi:10.3389/fphar.2022.832750 35273506 PMC8902503

[19] Mitchell G , Isberg RR . 2017. Innate immunity to intracellular pathogens: balancing microbial elimination and inflammation. Cell Host Microbe 22:166–175. doi:10.1016/j.chom.2017.07.005 28799902 PMC5562164

[20] Huang J , Brumell JH . 2014. Bacteria-autophagy interplay: a battle for survival. Nat Rev Microbiol 12:101–114. doi:10.1038/nrmicro3160 24384599 PMC7097477

[21] Shibutani ST , Saitoh T , Nowag H , Münz C , Yoshimori T . 2015. Autophagy and autophagy-related proteins in the immune system. Nat Immunol 16:1014–1024. doi:10.1038/ni.3273 26382870

[22] Bechelli J , Rumfield CS , Walker DH , Widen S , Khanipov K , Fang R . 2021. Subversion of host innate immunity by Rickettsia australis via a modified autophagic response in macrophages. Front Immunol 12:638469. doi:10.3389/fimmu.2021.638469 33912163 PMC8071864

[23] Bechelli J , Vergara L , Smalley C , Buzhdygan TP , Bender S , Zhang W , Liu Y , Popov VL , Wang J , Garg N , Hwang S , Walker DH , Fang R . 2019. ATG5 supports Rickettsia australis infection in macrophages in vitro and in vivo. Infect Immun 87:e00651-18. doi:10.1128/IAI.00651-18 PMC6300621 30297526

[24] Engström P , Burke TP , Mitchell G , Ingabire N , Mark KG , Golovkine G , Iavarone AT , Rape M , Cox JS , Welch MD . 2019. Evasion of autophagy mediated by Rickettsia surface protein OmpB is critical for virulence. Nat Microbiol 4:2538–2551. doi:10.1038/s41564-019-0583-6 31611642 PMC6988571

[25] Engström P , Burke TP , Tran CJ , Iavarone AT , Welch MD . 2021. Lysine methylation shields an intracellular pathogen from ubiquitylation and autophagy. Sci Adv 7:eabg2517. doi:10.1126/sciadv.abg2517 34172444 PMC8232902

[26] Rahman M.S , Ammerman NC , Sears KT , Ceraul SM , Azad AF . 2010. Functional characterization of a phospholipase A(2) homolog from Rickettsia typhi. J Bacteriol 192:3294–3303. doi:10.1128/JB.00155-10 20435729 PMC2897650

[27] Rennoll-Bankert KE , Rahman MS , Gillespie JJ , Guillotte ML , Kaur SJ , Lehman SS , Beier-Sexton M , Azad AF . 2015. Which way in? the RalF Arf-GEF orchestrates Rickettsia host cell invasion. PLoS Pathog 11:e1005115. doi:10.1371/journal.ppat.1005115 26291822 PMC4546372

[28] Rennoll-Bankert KE , Rahman MS , Guillotte ML , Lehman SS , Beier-Sexton M , Gillespie JJ , Azad AF . 2016. RalF-mediated activation of Arf6 controls Rickettsia typhi invasion by co-opting phosphoinositol metabolism. Infect Immun 84:3496–3506. doi:10.1128/IAI.00638-16 27698019 PMC5116726

[29] Rahman M. Sayeedur , Gillespie JJ , Kaur SJ , Sears KT , Ceraul SM , Beier-Sexton M , Azad AF , Isberg RR . 2013. Rickettsia typhi possesses phospholipase A2 enzymes that are involved in infection of host cells. PLoS Pathog 9:e1003399. doi:10.1371/journal.ppat.1003399 23818842 PMC3688537

[30] Voss OH , Gillespie JJ , Lehman SS , Rennoll SA , Beier-Sexton M , Rahman MS , Azad AF . 2020. Risk1, a phosphatidylinositol 3-kinase effector promotes Rickettsia typhi intracellular survival. mBio 11:e00820-20. doi:10.1128/mBio.00820-20 32546622 PMC7298712

[31] Lehman SS , Noriea NF , Aistleitner K , Clark TR , Dooley CA , Nair V , Kaur SJ , Rahman MS , Gillespie JJ , Azad AF , Hackstadt T . 2018. The rickettsial ankyrin repeat protein 2 is a type IV secreted effector that associates with the endoplasmic reticulum. mBio 9:e00975-18. doi:10.1128/mBio.00975-18 29946049 PMC6020290

[32] Voss OH , Cobb J , Gaytan H , Rivera Díaz N , Sanchez R , DeTolla L , Rahman MS , Azad AF . 2021. Pathogenic, but not nonpathogenic, Rickettsia spp. evade inflammasome-dependent IL-1 responses to establish an intracytosolic replication niche. mBio 13:e0291821. doi:10.1128/mbio.02918-21 35130729 PMC8822360

[33] Papp S , Moderzynski K , Rauch J , Heine L , Kuehl S , Richardt U , Mueller H , Fleischer B , Osterloh A . 2016. Liver necrosis and lethal systemic inflammation in a murine model of Rickettsia typhi infection: role of neutrophils, macrophages and NK cells. PLoS Negl Trop Dis 10:e0004935. doi:10.1371/journal.pntd.0004935 27548618 PMC4993389

[34] Burke TP , Engström P , Chavez RA , Fonbuena JA , Vance RE , Welch MD . 2020. Inflammasome-mediated antagonism of type I interferon enhances Rickettsia pathogenesis. Nat Microbiol 5:688–696. doi:10.1038/s41564-020-0673-5 32123346 PMC7239376

[35] Smalley C , Bechelli J , Rockx-Brouwer D , Saito T , Azar SR , Ismail N , Walker DH , Fang R , Winslow GM . 2016. Rickettsia australis activates inflammasome in human and murine macrophages. PLoS ONE 11:e0157231. doi:10.1371/journal.pone.0157231 27362650 PMC4928923

[36] Kristof MN , Allen PE , Yutzy LD , Thibodaux B , Paddock CD , Martinez JJ . 2021. Significant growth by Rickettsia species within human macrophage-like cells is a phenotype correlated with the ability to cause disease in mammals. Pathogens 10:1–14. doi:10.3390/pathogens10020228 PMC7934685 33669499

[37] Curto P , Simões I , Riley SP , Martinez JJ . 2016. Differences in intracellular fate of two spotted fever group Rickettsia in macrophage-like cells. Front Cell Infect Microbiol 6:80. doi:10.3389/fcimb.2016.00080 27525249 PMC4965480

[38] Klionsky DJ , Abdelmohsen K , Abe A , Abedin MJ , Abeliovich H , Acevedo Arozena A , Adachi H , Adams CM , Adams PD , Adeli K , Adhihetty PJ , Adler SG , Agam G , Agarwal R , Aghi MK , Agnello M , Agostinis P , Aguilar PV , Aguirre-Ghiso J , Airoldi EM , Ait-Si-Ali S , Akematsu T , Akporiaye ET , Al-Rubeai M , Albaiceta GM , Albanese C , Albani D , Albert ML , Aldudo J , Algül H , Alirezaei M , Alloza I , Almasan A , Almonte-Beceril M , Alnemri ES , Alonso C , Altan-Bonnet N , Altieri DC , Alvarez S , Alvarez-Erviti L , Alves S , Amadoro G , Amano A , Amantini C , Ambrosio S , Amelio I , Amer AO , Amessou M , Amon A , An Z , Anania FA , Andersen SU , Andley UP , Andreadi CK , Andrieu-Abadie N , Anel A , Ann DK , Anoopkumar-Dukie S , Antonioli M , Aoki H , Apostolova N , Aquila S , Aquilano K , Araki K , Arama E , Aranda A , Araya J , Arcaro A , Arias E , Arimoto H , Ariosa AR , Armstrong JL , Arnould T , Arsov I , Asanuma K , Askanas V , Asselin E , Atarashi R , Atherton SS , Atkin JD , Attardi LD , Auberger P , Auburger G , Aurelian L , Autelli R , Avagliano L , Avantaggiati ML , Avrahami L , Awale S , Azad N , Bachetti T , Backer JM , Bae D-H , Bae J-S , Bae O-N , Bae SH , Baehrecke EH , Baek S-H , Baghdiguian S , Bagniewska-Zadworna A , Bai H , Bai J , Bai X-Y , Bailly Y , Balaji KN , Balduini W , Ballabio A , Balzan R , Banerjee R , Bánhegyi G , Bao H , Barbeau B , Barrachina MD , Barreiro E , Bartel B , Bartolomé A , Bassham DC , Bassi MT , Bast RC , Basu A , Batista MT , Batoko H , Battino M , Bauckman K , Baumgarner BL , Bayer KU , Beale R , Beaulieu J-F , Beck GR , Becker C , Beckham JD , Bédard P-A , Bednarski PJ , Begley TJ , Behl C , Behrends C , Behrens GM , Behrns KE , Bejarano E , Belaid A , Belleudi F , Bénard G , Berchem G , Bergamaschi D , Bergami M , Berkhout B , Berliocchi L , Bernard A , Bernard M , Bernassola F , Bertolotti A , Bess AS , Besteiro S , Bettuzzi S , Bhalla S , Bhattacharyya S , Bhutia SK , Biagosch C , Bianchi MW , Biard-Piechaczyk M , Billes V , Bincoletto C , Bingol B , Bird SW , Bitoun M , Bjedov I , Blackstone C , Blanc L , Blanco GA , Blomhoff HK , Boada-Romero E , Böckler S , Boes M , Boesze-Battaglia K , Boise LH , Bolino A , Boman A , Bonaldo P , Bordi M , Bosch J , Botana LM , Botti J , Bou G , Bouché M , Bouchecareilh M , Boucher M-J , Boulton ME , Bouret SG , Boya P , Boyer-Guittaut M , Bozhkov PV , Brady N , Braga VM , Brancolini C , Braus GH , Bravo-San Pedro JM , Brennan LA , Bresnick EH , Brest P , Bridges D , Bringer M-A , Brini M , Brito GC , Brodin B , Brookes PS , Brown EJ , Brown K , Broxmeyer HE , Bruhat A , Brum PC , Brumell JH , Brunetti-Pierri N , Bryson-Richardson RJ , Buch S , Buchan AM , Budak H , Bulavin DV , Bultman SJ , Bultynck G , Bumbasirevic V , Burelle Y , Burke RE , Burmeister M , Bütikofer P , Caberlotto L , Cadwell K , Cahova M , Cai D , Cai J , Cai Q , Calatayud S , Camougrand N , Campanella M , Campbell GR , Campbell M , Campello S , Candau R , Caniggia I , Cantoni L , Cao L , Caplan AB , Caraglia M , Cardinali C , Cardoso SM , Carew JS , Carleton LA , Carlin CR , Carloni S , Carlsson SR , Carmona-Gutierrez D , Carneiro LA , Carnevali O , Carra S , Carrier A , Carroll B , Casas C , Casas J , Cassinelli G , Castets P , Castro-Obregon S , Cavallini G , Ceccherini I , Cecconi F , Cederbaum AI , Ceña V , Cenci S , Cerella C , Cervia D , Cetrullo S , Chaachouay H , Chae H-J , Chagin AS , Chai C-Y , Chakrabarti G , Chamilos G , Chan EY , Chan MT , Chandra D , Chandra P , Chang C-P , Chang RC-C , Chang TY , Chatham JC , Chatterjee S , Chauhan S , Che Y , Cheetham ME , Cheluvappa R , Chen C-J , Chen G , Chen G-C , Chen G , Chen H , Chen JW , Chen J-K , Chen M , Chen M , Chen P , Chen Q , Chen Q , Chen S-D , Chen S , Chen SS-L , Chen W , Chen W-J , Chen WQ , Chen W , Chen X , Chen Y-H , Chen Y-G , Chen Y , Chen Y , Chen Y , Chen Y-J , Chen Y-Q , Chen Y , Chen Z , Chen Z , Cheng A , Cheng CH , Cheng H , Cheong H , Cherry S , Chesney J , Cheung CHA , Chevet E , Chi HC , Chi S-G , Chiacchiera F , Chiang H-L , Chiarelli R , Chiariello M , Chieppa M , Chin L-S , Chiong M , Chiu GN , Cho D-H , Cho S-G , Cho WC , Cho Y-Y , Cho Y-S , Choi AM , Choi E-J , Choi E-K , Choi J , Choi ME , Choi S-I , Chou T-F , Chouaib S , Choubey D , Choubey V , Chow K-C , Chowdhury K , Chu CT , Chuang T-H , Chun T , Chung H , Chung T , Chung Y-L , Chwae Y-J , Cianfanelli V , Ciarcia R , Ciechomska IA , Ciriolo MR , Cirone M , Claerhout S , Clague MJ , Clària J , Clarke PG , Clarke R , Clementi E , Cleyrat C , Cnop M , Coccia EM , Cocco T , Codogno P , Coers J , Cohen EE , Colecchia D , Coletto L , Coll NS , Colucci-Guyon E , Comincini S , Condello M , Cook KL , Coombs GH , Cooper CD , Cooper JM , Coppens I , Corasaniti MT , Corazzari M , Corbalan R , Corcelle-Termeau E , Cordero MD , Corral-Ramos C , Corti O , Cossarizza A , Costelli P , Costes S , Cotman SL , Coto-Montes A , Cottet S , Couve E , Covey LR , Cowart LA , Cox JS , Coxon FP , Coyne CB , Cragg MS , Craven RJ , Crepaldi T , Crespo JL , Criollo A , Crippa V , Cruz MT , Cuervo AM , Cuezva JM , Cui T , Cutillas PR , Czaja MJ , Czyzyk-Krzeska MF , Dagda RK , Dahmen U , Dai C , Dai W , Dai Y , Dalby KN , Dalla Valle L , Dalmasso G , D’Amelio M , Damme M , Darfeuille-Michaud A , Dargemont C , Darley-Usmar VM , Dasarathy S , Dasgupta B , Dash S , Dass CR , Davey HM , Davids LM , Dávila D , Davis RJ , Dawson TM , Dawson VL , Daza P , de Belleroche J , de Figueiredo P , de Figueiredo RCBQ , de la Fuente J , De Martino L , De Matteis A , De Meyer GR , De Milito A , De Santi M , de Souza W , De Tata V , De Zio D , Debnath J , Dechant R , Decuypere J-P , Deegan S , Dehay B , Del Bello B , Del Re DP , Delage-Mourroux R , Delbridge LM , Deldicque L , Delorme-Axford E , Deng Y , Dengjel J , Denizot M , Dent P , Der CJ , Deretic V , Derrien B , Deutsch E , Devarenne TP , Devenish RJ , Di Bartolomeo S , Di Daniele N , Di Domenico F , Di Nardo A , Di Paola S , Di Pietro A , Di Renzo L , DiAntonio A , Díaz-Araya G , Díaz-Laviada I , Diaz-Meco MT , Diaz-Nido J , Dickey CA , Dickson RC , Diederich M , Digard P , Dikic I , Dinesh-Kumar SP , Ding C , Ding W-X , Ding Z , Dini L , Distler JH , Diwan A , Djavaheri-Mergny M , Dmytruk K , Dobson RC , Doetsch V , Dokladny K , Dokudovskaya S , Donadelli M , Dong XC , Dong X , Dong Z , Donohue TM , Doran KS , D’Orazi G , Dorn GW , Dosenko V , Dridi S , Drucker L , Du J , Du L-L , Du L , du Toit A , Dua P , Duan L , Duann P , Dubey VK , Duchen MR , Duchosal MA , Duez H , Dugail I , Dumit VI , Duncan MC , Dunlop EA , Dunn WA , Dupont N , Dupuis L , Durán RV , Durcan TM , Duvezin-Caubet S , Duvvuri U , Eapen V , Ebrahimi-Fakhari D , Echard A , Eckhart L , Edelstein CL , Edinger AL , Eichinger L , Eisenberg T , Eisenberg-Lerner A , Eissa NT , El-Deiry WS , El-Khoury V , Elazar Z , Eldar-Finkelman H , Elliott CJ , Emanuele E , Emmenegger U , Engedal N , Engelbrecht A-M , Engelender S , Enserink JM , Erdmann R , Erenpreisa J , Eri R , Eriksen JL , Erman A , Escalante R , Eskelinen E-L , Espert L , Esteban-Martínez L , Evans TJ , Fabri M , Fabrias G , Fabrizi C , Facchiano A , Færgeman NJ , Faggioni A , Fairlie WD , Fan C , Fan D , Fan J , Fang S , Fanto M , Fanzani A , Farkas T , Faure M , Favier FB , Fearnhead H , Federici M , Fei E , Felizardo TC , Feng H , Feng Y , Feng Y , Ferguson TA , Fernández ÁF , Fernandez-Barrena MG , Fernandez-Checa JC , Fernández-López A , Fernandez-Zapico ME , Feron O , Ferraro E , Ferreira-Halder CV , Fesus L , Feuer R , Fiesel FC , Filippi-Chiela EC , Filomeni G , Fimia GM , Fingert JH , Finkbeiner S , Finkel T , Fiorito F , Fisher PB , Flajolet M , Flamigni F , Florey O , Florio S , Floto RA , Folini M , Follo C , Fon EA , Fornai F , Fortunato F , Fraldi A , Franco R , Francois A , François A , Frankel LB , Fraser ID , Frey N , Freyssenet DG , Frezza C , Friedman SL , Frigo DE , Fu D , Fuentes JM , Fueyo J , Fujitani Y , Fujiwara Y , Fujiya M , Fukuda M , Fulda S , Fusco C , Gabryel B , Gaestel M , Gailly P , Gajewska M , Galadari S , Galili G , Galindo I , Galindo MF , Galliciotti G , Galluzzi L , Galluzzi L , Galy V , Gammoh N , Gandy S , Ganesan AK , Ganesan S , Ganley IG , Gannagé M , Gao F-B , Gao F , Gao J-X , García Nannig L , García Véscovi E , Garcia-Macía M , Garcia-Ruiz C , Garg AD , Garg PK , Gargini R , Gassen NC , Gatica D , Gatti E , Gavard J , Gavathiotis E , Ge L , Ge P , Ge S , Gean P-W , Gelmetti V , Genazzani AA , Geng J , Genschik P , Gerner L , Gestwicki JE , Gewirtz DA , Ghavami S , Ghigo E , Ghosh D , Giammarioli AM , Giampieri F , Giampietri C , Giatromanolaki A , Gibbings DJ , Gibellini L , Gibson SB , Ginet V , Giordano A , Giorgini F , Giovannetti E , Girardin SE , Gispert S , Giuliano S , Gladson CL , Glavic A , Gleave M , Godefroy N , Gogal RM , Gokulan K , Goldman GH , Goletti D , Goligorsky MS , Gomes AV , Gomes LC , Gomez H , Gomez-Manzano C , Gómez-Sánchez R , Gonçalves DA , Goncu E , Gong Q , Gongora C , Gonzalez CB , Gonzalez-Alegre P , Gonzalez-Cabo P , González-Polo RA , Goping IS , Gorbea C , Gorbunov NV , Goring DR , Gorman AM , Gorski SM , Goruppi S , Goto-Yamada S , Gotor C , Gottlieb RA , Gozes I , Gozuacik D , Graba Y , Graef M , Granato GE , Grant GD , Grant S , Gravina GL , Green DR , Greenhough A , Greenwood MT , Grimaldi B , Gros F , Grose C , Groulx J-F , Gruber F , Grumati P , Grune T , Guan J-L , Guan K-L , Guerra B , Guillen C , Gulshan K , Gunst J , Guo C , Guo L , Guo M , Guo W , Guo X-G , Gust AA , Gustafsson ÅB , Gutierrez E , Gutierrez MG , Gwak H-S , Haas A , Haber JE , Hadano S , Hagedorn M , Hahn DR , Halayko AJ , Hamacher-Brady A , Hamada K , Hamai A , Hamann A , Hamasaki M , Hamer I , Hamid Q , Hammond EM , Han F , Han W , Handa JT , Hanover JA , Hansen M , Harada M , Harhaji-Trajkovic L , Harper JW , Harrath AH , Harris AL , Harris J , Hasler U , Hasselblatt P , Hasui K , Hawley RG , Hawley TS , He C , He CY , He F , He G , He R-R , He X-H , He Y-W , He Y-Y , Heath JK , Hébert M-J , Heinzen RA , Helgason GV , Hensel M , Henske EP , Her C , Herman PK , Hernández A , Hernandez C , Hernández-Tiedra S , Hetz C , Hiesinger PR , Higaki K , Hilfiker S , Hill BG , Hill JA , Hill WD , Hino K , Hofius D , Hofman P , Höglinger GU , Höhfeld J , Holz MK , Hong Y , Hood DA , Hoozemans JJ , Hoppe T , Hsu C , Hsu C-Y , Hsu L-C , Hu D , Hu G , Hu H-M , Hu H , Hu MC , Hu Y-C , Hu Z-W , Hua F , Hua Y , Huang C , Huang H-L , Huang K-H , Huang K-Y , Huang S , Huang S , Huang W-P , Huang Y-R , Huang Y , Huang Y , Huber TB , Huebbe P , Huh W-K , Hulmi JJ , Hur GM , Hurley JH , Husak Z , Hussain SN , Hussain S , Hwang JJ , Hwang S , Hwang TI , Ichihara A , Imai Y , Imbriano C , Inomata M , Into T , Iovane V , Iovanna JL , Iozzo RV , Ip NY , Irazoqui JE , Iribarren P , Isaka Y , Isakovic AJ , Ischiropoulos H , Isenberg JS , Ishaq M , Ishida H , Ishii I , Ishmael JE , Isidoro C , Isobe K-I , Isono E , Issazadeh-Navikas S , Itahana K , Itakura E , Ivanov AI , Iyer AKV , Izquierdo JM , Izumi Y , Izzo V , Jäättelä M , Jaber N , Jackson DJ , Jackson WT , Jacob TG , Jacques TS , Jagannath C , Jain A , Jana NR , Jang BK , Jani A , Janji B , Jannig PR , Jansson PJ , Jean S , Jendrach M , Jeon J-H , Jessen N , Jeung E-B , Jia K , Jia L , Jiang H , Jiang H , Jiang L , Jiang T , Jiang X , Jiang X , Jiang X , Jiang Y , Jiang Y , Jiménez A , Jin C , Jin H , Jin L , Jin M , Jin S , Jinwal UK , Jo E-K , Johansen T , Johnson DE , Johnson GV , Johnson JD , Jonasch E , Jones C , Joosten LA , Jordan J , Joseph A-M , Joseph B , Joubert AM , Ju D , Ju J , Juan H-F , Juenemann K , Juhász G , Jung HS , Jung JU , Jung Y-K , Jungbluth H , Justice MJ , Jutten B , Kaakoush NO , Kaarniranta K , Kaasik A , Kabuta T , Kaeffer B , Kågedal K , Kahana A , Kajimura S , Kakhlon O , Kalia M , Kalvakolanu DV , Kamada Y , Kambas K , Kaminskyy VO , Kampinga HH , Kandouz M , Kang C , Kang R , Kang T-C , Kanki T , Kanneganti T-D , Kanno H , Kanthasamy AG , Kantorow M , Kaparakis-Liaskos M , Kapuy O , Karantza V , Karim MR , Karmakar P , Kaser A , Kaushik S , Kawula T , Kaynar AM , Ke P-Y , Ke Z-J , Kehrl JH , Keller KE , Kemper JK , Kenworthy AK , Kepp O , Kern A , Kesari S , Kessel D , Ketteler R , Kettelhut I do C , Khambu B , Khan MM , Khandelwal VK , Khare S , Kiang JG , Kiger AA , Kihara A , Kim AL , Kim CH , Kim DR , Kim D-H , Kim EK , Kim HY , Kim H-R , Kim J-S , Kim JH , Kim JC , Kim JH , Kim KW , Kim MD , Kim M-M , Kim PK , Kim SW , Kim S-Y , Kim Y-S , Kim Y , Kimchi A , Kimmelman AC , Kimura T , King JS , Kirkegaard K , Kirkin V , Kirshenbaum LA , Kishi S , Kitajima Y , Kitamoto K , Kitaoka Y , Kitazato K , Kley RA , Klimecki WT , Klinkenberg M , Klucken J , Knævelsrud H , Knecht E , Knuppertz L , Ko J-L , Kobayashi S , Koch JC , Koechlin-Ramonatxo C , Koenig U , Koh YH , Köhler K , Kohlwein SD , Koike M , Komatsu M , Kominami E , Kong D , Kong HJ , Konstantakou EG , Kopp BT , Korcsmaros T , Korhonen L , Korolchuk VI , Koshkina NV , Kou Y , Koukourakis MI , Koumenis C , Kovács AL , Kovács T , Kovacs WJ , Koya D , Kraft C , Krainc D , Kramer H , Kravic-Stevovic T , Krek W , Kretz-Remy C , Krick R , Krishnamurthy M , Kriston-Vizi J , Kroemer G , Kruer MC , Kruger R , Ktistakis NT , Kuchitsu K , Kuhn C , Kumar AP , Kumar A , Kumar A , Kumar D , Kumar D , Kumar R , Kumar S , Kundu M , Kung H-J , Kuno A , Kuo S-H , Kuret J , Kurz T , Kwok T , Kwon TK , Kwon YT , Kyrmizi I , La Spada AR , Lafont F , Lahm T , Lakkaraju A , Lam T , Lamark T , Lancel S , Landowski TH , Lane DJR , Lane JD , Lanzi C , Lapaquette P , Lapierre LR , Laporte J , Laukkarinen J , Laurie GW , Lavandero S , Lavie L , LaVoie MJ , Law BYK , Law HK-W , Law KB , Layfield R , Lazo PA , Le Cam L , Le Roch KG , Le Stunff H , Leardkamolkarn V , Lecuit M , Lee B-H , Lee C-H , Lee EF , Lee GM , Lee H-J , Lee H , Lee JK , Lee J , Lee J-H , Lee JH , Lee M , Lee M-S , Lee PJ , Lee SW , Lee S-J , Lee S-J , Lee SY , Lee SH , Lee SS , Lee S-J , Lee S , Lee Y-R , Lee YJ , Lee YH , Leeuwenburgh C , Lefort S , Legouis R , Lei J , Lei Q-Y , Leib DA , Leibowitz G , Lekli I , Lemaire SD , Lemasters JJ , Lemberg MK , Lemoine A , Leng S , Lenz G , Lenzi P , Lerman LO , Lettieri Barbato D , Leu JI-J , Leung HY , Levine B , Lewis PA , Lezoualc’h F , Li C , Li F , Li F-J , Li J , Li K , Li L , Li M , Li M , Li Q , Li R , Li S , Li W , Li W , Li X , Li Y , Lian J , Liang C , Liang Q , Liao Y , Liberal J , Liberski PP , Lie P , Lieberman AP , Lim HJ , Lim K-L , Lim K , Lima RT , Lin C-S , Lin C-F , Lin F , Lin F , Lin F-C , Lin K , Lin K-H , Lin P-H , Lin T , Lin W-W , Lin Y-S , Lin Y , Linden R , Lindholm D , Lindqvist LM , Lingor P , Linkermann A , Liotta LA , Lipinski MM , Lira VA , Lisanti MP , Liton PB , Liu B , Liu C , Liu C-F , Liu F , Liu H-J , Liu J , Liu J-J , Liu J-L , Liu K , Liu L , Liu L , Liu Q , Liu R-Y , Liu S , Liu S , Liu W , Liu X-D , Liu X , Liu X-H , Liu X , Liu X , Liu X , Liu Y , Liu Y , Liu Z , Liu Z , Liuzzi JP , Lizard G , Ljujic M , Lodhi IJ , Logue SE , Lokeshwar BL , Long YC , Lonial S , Loos B , López-Otín C , López-Vicario C , Lorente M , Lorenzi PL , Lõrincz P , Los M , Lotze MT , Lovat PE , Lu B , Lu B , Lu J , Lu Q , Lu S-M , Lu S , Lu Y , Luciano F , Luckhart S , Lucocq JM , Ludovico P , Lugea A , Lukacs NW , Lum JJ , Lund AH , Luo H , Luo J , Luo S , Luparello C , Lyons T , Ma J , Ma Y , Ma Y , Ma Z , Machado J , Machado-Santelli GM , Macian F , MacIntosh GC , MacKeigan JP , Macleod KF , MacMicking JD , MacMillan-Crow LA , Madeo F , Madesh M , Madrigal-Matute J , Maeda A , Maeda T , Maegawa G , Maellaro E , Maes H , Magariños M , Maiese K , Maiti TK , Maiuri L , Maiuri MC , Maki CG , Malli R , Malorni W , Maloyan A , Mami-Chouaib F , Man N , Mancias JD , Mandelkow E-M , Mandell MA , Manfredi AA , Manié SN , Manzoni C , Mao K , Mao Z , Mao Z-W , Marambaud P , Marconi AM , Marelja Z , Marfe G , Margeta M , Margittai E , Mari M , Mariani FV , Marin C , Marinelli S , Mariño G , Markovic I , Marquez R , Martelli AM , Martens S , Martin KR , Martin SJ , Martin S , Martin-Acebes MA , Martín-Sanz P , Martinand-Mari C , Martinet W , Martinez J , Martinez-Lopez N , Martinez-Outschoorn U , Martínez-Velázquez M , Martinez-Vicente M , Martins WK , Mashima H , Mastrianni JA , Matarese G , Matarrese P , Mateo R , Matoba S , Matsumoto N , Matsushita T , Matsuura A , Matsuzawa T , Mattson MP , Matus S , Maugeri N , Mauvezin C , Mayer A , Maysinger D , Mazzolini GD , McBrayer MK , McCall K , McCormick C , McInerney GM , McIver SC , McKenna S , McMahon JJ , McNeish IA , Mechta-Grigoriou F , Medema JP , Medina DL , Megyeri K , Mehrpour M , Mehta JL , Mei Y , Meier U-C , Meijer AJ , Meléndez A , Melino G , Melino S , de Melo EJT , Mena MA , Meneghini MD , Menendez JA , Menezes R , Meng L , Meng L-H , Meng S , Menghini R , Menko AS , Menna-Barreto RF , Menon MB , Meraz-Ríos MA , Merla G , Merlini L , Merlot AM , Meryk A , Meschini S , Meyer JN , Mi M-T , Miao C-Y , Micale L , Michaeli S , Michiels C , Migliaccio AR , Mihailidou AS , Mijaljica D , Mikoshiba K , Milan E , Miller-Fleming L , Mills GB , Mills IG , Minakaki G , Minassian BA , Ming X-F , Minibayeva F , Minina EA , Mintern JD , Minucci S , Miranda-Vizuete A , Mitchell CH , Miyamoto S , Miyazawa K , Mizushima N , Mnich K , Mograbi B , Mohseni S , Moita LF , Molinari M , Molinari M , Møller AB , Mollereau B , Mollinedo F , Mongillo M , Monick MM , Montagnaro S , Montell C , Moore DJ , Moore MN , Mora-Rodriguez R , Moreira PI , Morel E , Morelli MB , Moreno S , Morgan MJ , Moris A , Moriyasu Y , Morrison JL , Morrison LA , Morselli E , Moscat J , Moseley PL , Mostowy S , Motori E , Mottet D , Mottram JC , Moussa CE-H , Mpakou VE , Mukhtar H , Mulcahy Levy JM , Muller S , Muñoz-Moreno R , Muñoz-Pinedo C , Münz C , Murphy ME , Murray JT , Murthy A , Mysorekar IU , Nabi IR , Nabissi M , Nader GA , Nagahara Y , Nagai Y , Nagata K , Nagelkerke A , Nagy P , Naidu SR , Nair S , Nakano H , Nakatogawa H , Nanjundan M , Napolitano G , Naqvi NI , Nardacci R , Narendra DP , Narita M , Nascimbeni AC , Natarajan R , Navegantes LC , Nawrocki ST , Nazarko TY , Nazarko VY , Neill T , Neri LM , Netea MG , Netea-Maier RT , Neves BM , Ney PA , Nezis IP , Nguyen HT , Nguyen HP , Nicot A-S , Nilsen H , Nilsson P , Nishimura M , Nishino I , Niso-Santano M , Niu H , Nixon RA , Njar VC , Noda T , Noegel AA , Nolte EM , Norberg E , Norga KK , Noureini SK , Notomi S , Notterpek L , Nowikovsky K , Nukina N , Nürnberger T , O’Donnell VB , O’Donovan T , O’Dwyer PJ , Oehme I , Oeste CL , Ogawa M , Ogretmen B , Ogura Y , Oh YJ , Ohmuraya M , Ohshima T , Ojha R , Okamoto K , Okazaki T , Oliver FJ , Ollinger K , Olsson S , Orban DP , Ordonez P , Orhon I , Orosz L , O’Rourke EJ , Orozco H , Ortega AL , Ortona E , Osellame LD , Oshima J , Oshima S , Osiewacz HD , Otomo T , Otsu K , Ou J-HJ , Outeiro TF , Ouyang D-Y , Ouyang H , Overholtzer M , Ozbun MA , Ozdinler PH , Ozpolat B , Pacelli C , Paganetti P , Page G , Pages G , Pagnini U , Pajak B , Pak SC , Pakos-Zebrucka K , Pakpour N , Palková Z , Palladino F , Pallauf K , Pallet N , Palmieri M , Paludan SR , Palumbo C , Palumbo S , Pampliega O , Pan H , Pan W , Panaretakis T , Pandey A , Pantazopoulou A , Papackova Z , Papademetrio DL , Papassideri I , Papini A , Parajuli N , Pardo J , Parekh VV , Parenti G , Park J-I , Park J , Park OK , Parker R , Parlato R , Parys JB , Parzych KR , Pasquet J-M , Pasquier B , Pasumarthi KB , Patschan D , Patterson C , Pattingre S , Pattison S , Pause A , Pavenstädt H , Pavone F , Pedrozo Z , Peña FJ , Peñalva MA , Pende M , Peng J , Penna F , Penninger JM , Pensalfini A , Pepe S , Pereira GJ , Pereira PC , Pérez-de la Cruz V , Pérez-Pérez ME , Pérez-Rodríguez D , Pérez-Sala D , Perier C , Perl A , Perlmutter DH , Perrotta I , Pervaiz S , Pesonen M , Pessin JE , Peters GJ , Petersen M , Petrache I , Petrof BJ , Petrovski G , Phang JM , Piacentini M , Pierdominici M , Pierre P , Pierrefite-Carle V , Pietrocola F , Pimentel-Muiños FX , Pinar M , Pineda B , Pinkas-Kramarski R , Pinti M , Pinton P , Piperdi B , Piret JM , Platanias LC , Platta HW , Plowey ED , Pöggeler S , Poirot M , Polčic P , Poletti A , Poon AH , Popelka H , Popova B , Poprawa I , Poulose SM , Poulton J , Powers SK , Powers T , Pozuelo-Rubio M , Prak K , Prange R , Prescott M , Priault M , Prince S , Proia RL , Proikas-Cezanne T , Prokisch H , Promponas VJ , Przyklenk K , Puertollano R , Pugazhenthi S , Puglielli L , Pujol A , Puyal J , Pyeon D , Qi X , Qian W-B , Qin Z-H , Qiu Y , Qu Z , Quadrilatero J , Quinn F , Raben N , Rabinowich H , Radogna F , Ragusa MJ , Rahmani M , Raina K , Ramanadham S , Ramesh R , Rami A , Randall-Demllo S , Randow F , Rao H , Rao VA , Rasmussen BB , Rasse TM , Ratovitski EA , Rautou P-E , Ray SK , Razani B , Reed BH , Reggiori F , Rehm M , Reichert AS , Rein T , Reiner DJ , Reits E , Ren J , Ren X , Renna M , Reusch JE , Revuelta JL , Reyes L , Rezaie AR , Richards RI , Richardson DR , Richetta C , Riehle MA , Rihn BH , Rikihisa Y , Riley BE , Rimbach G , Rippo MR , Ritis K , Rizzi F , Rizzo E , Roach PJ , Robbins J , Roberge M , Roca G , Roccheri MC , Rocha S , Rodrigues CMP , Rodríguez CI , de Cordoba SR , Rodriguez-Muela N , Roelofs J , Rogov VV , Rohn TT , Rohrer B , Romanelli D , Romani L , Romano PS , Roncero MIG , Rosa JL , Rosello A , Rosen KV , Rosenstiel P , Rost-Roszkowska M , Roth KA , Roué G , Rouis M , Rouschop KM , Ruan DT , Ruano D , Rubinsztein DC , Rucker EB , Rudich A , Rudolf E , Rudolf R , Ruegg MA , Ruiz-Roldan C , Ruparelia AA , Rusmini P , Russ DW , Russo GL , Russo G , Russo R , Rusten TE , Ryabovol V , Ryan KM , Ryter SW , Sabatini DM , Sacher M , Sachse C , Sack MN , Sadoshima J , Saftig P , Sagi-Eisenberg R , Sahni S , Saikumar P , Saito T , Saitoh T , Sakakura K , Sakoh-Nakatogawa M , Sakuraba Y , Salazar-Roa M , Salomoni P , Saluja AK , Salvaterra PM , Salvioli R , Samali A , Sanchez AM , Sánchez-Alcázar JA , Sanchez-Prieto R , Sandri M , Sanjuan MA , Santaguida S , Santambrogio L , Santoni G , Dos Santos CN , Saran S , Sardiello M , Sargent G , Sarkar P , Sarkar S , Sarrias MR , Sarwal MM , Sasakawa C , Sasaki M , Sass M , Sato K , Sato M , Satriano J , Savaraj N , Saveljeva S , Schaefer L , Schaible UE , Scharl M , Schatzl HM , Schekman R , Scheper W , Schiavi A , Schipper HM , Schmeisser H , Schmidt J , Schmitz I , Schneider BE , Schneider EM , Schneider JL , Schon EA , Schönenberger MJ , Schönthal AH , Schorderet DF , Schröder B , Schuck S , Schulze RJ , Schwarten M , Schwarz TL , Sciarretta S , Scotto K , Scovassi AI , Screaton RA , Screen M , Seca H , Sedej S , Segatori L , Segev N , Seglen PO , Seguí-Simarro JM , Segura-Aguilar J , Seki E , Sell C , Seiliez I , Semenkovich CF , Semenza GL , Sen U , Serra AL , Serrano-Puebla A , Sesaki H , Setoguchi T , Settembre C , Shacka JJ , Shajahan-Haq AN , Shapiro IM , Sharma S , She H , Shen C-KJ , Shen C-C , Shen H-M , Shen S , Shen W , Sheng R , Sheng X , Sheng Z-H , Shepherd TG , Shi J , Shi Q , Shi Q , Shi Y , Shibutani S , Shibuya K , Shidoji Y , Shieh J-J , Shih C-M , Shimada Y , Shimizu S , Shin DW , Shinohara ML , Shintani M , Shintani T , Shioi T , Shirabe K , Shiri-Sverdlov R , Shirihai O , Shore GC , Shu C-W , Shukla D , Sibirny AA , Sica V , Sigurdson CJ , Sigurdsson EM , Sijwali PS , Sikorska B , Silveira WA , Silvente-Poirot S , Silverman GA , Simak J , Simmet T , Simon AK , Simon H-U , Simone C , Simons M , Simonsen A , Singh R , Singh SV , Singh SK , Sinha D , Sinha S , Sinicrope FA , Sirko A , Sirohi K , Sishi BJ , Sittler A , Siu PM , Sivridis E , Skwarska A , Slack R , Slaninová I , Slavov N , Smaili SS , Smalley KS , Smith DR , Soenen SJ , Soleimanpour SA , Solhaug A , Somasundaram K , Son JH , Sonawane A , Song C , Song F , Song HK , Song J-X , Song W , Soo KY , Sood AK , Soong TW , Soontornniyomkij V , Sorice M , Sotgia F , Soto-Pantoja DR , Sotthibundhu A , Sousa MJ , Spaink HP , Span PN , Spang A , Sparks JD , Speck PG , Spector SA , Spies CD , Springer W , Clair DS , Stacchiotti A , Staels B , Stang MT , Starczynowski DT , Starokadomskyy P , Steegborn C , Steele JW , Stefanis L , Steffan J , Stellrecht CM , Stenmark H , Stepkowski TM , Stern ST , Stevens C , Stockwell BR , Stoka V , Storchova Z , Stork B , Stratoulias V , Stravopodis DJ , Strnad P , Strohecker AM , Ström A-L , Stromhaug P , Stulik J , Su Y-X , Su Z , Subauste CS , Subramaniam S , Sue CM , Suh SW , Sui X , Sukseree S , Sulzer D , Sun F-L , Sun J , Sun J , Sun S-Y , Sun Y , Sun Y , Sun Y , Sundaramoorthy V , Sung J , Suzuki H , Suzuki K , Suzuki N , Suzuki T , Suzuki YJ , Swanson MS , Swanton C , Swärd K , Swarup G , Sweeney ST , Sylvester PW , Szatmari Z , Szegezdi E , Szlosarek PW , Taegtmeyer H , Tafani M , Taillebourg E , Tait SW , Takacs-Vellai K , Takahashi Y , Takáts S , Takemura G , Takigawa N , Talbot NJ , Tamagno E , Tamburini J , Tan C-P , Tan L , Tan ML , Tan M , Tan Y-J , Tanaka K , Tanaka M , Tang D , Tang D , Tang G , Tanida I , Tanji K , Tannous BA , Tapia JA , Tasset-Cuevas I , Tatar M , Tavassoly I , Tavernarakis N , Taylor A , Taylor GS , Taylor GA , Taylor JP , Taylor MJ , Tchetina EV , Tee AR , Teixeira-Clerc F , Telang S , Tencomnao T , Teng B-B , Teng R-J , Terro F , Tettamanti G , Theiss AL , Theron AE , Thomas KJ , Thomé MP , Thomes PG , Thorburn A , Thorner J , Thum T , Thumm M , Thurston TL , Tian L , Till A , Ting JP-Y , Titorenko VI , Toker L , Toldo S , Tooze SA , Topisirovic I , Torgersen ML , Torosantucci L , Torriglia A , Torrisi MR , Tournier C , Towns R , Trajkovic V , Travassos LH , Triola G , Tripathi DN , Trisciuoglio D , Troncoso R , Trougakos IP , Truttmann AC , Tsai K-J , Tschan MP , Tseng Y-H , Tsukuba T , Tsung A , Tsvetkov AS , Tu S , Tuan H-Y , Tucci M , Tumbarello DA , Turk B , Turk V , Turner RF , Tveita AA , Tyagi SC , Ubukata M , Uchiyama Y , Udelnow A , Ueno T , Umekawa M , Umemiya-Shirafuji R , Underwood BR , Ungermann C , Ureshino RP , Ushioda R , Uversky VN , Uzcátegui NL , Vaccari T , Vaccaro MI , Váchová L , Vakifahmetoglu-Norberg H , Valdor R , Valente EM , Vallette F , Valverde AM , Van den Berghe G , Van Den Bosch L , van den Brink GR , van der Goot FG , van der Klei IJ , van der Laan LJ , van Doorn WG , van Egmond M , van Golen KL , Van Kaer L , van Lookeren Campagne M , Vandenabeele P , Vandenberghe W , Vanhorebeek I , Varela-Nieto I , Vasconcelos MH , Vasko R , Vavvas DG , Vega-Naredo I , Velasco G , Velentzas AD , Velentzas PD , Vellai T , Vellenga E , Vendelbo MH , Venkatachalam K , Ventura N , Ventura S , Veras PS , Verdier M , Vertessy BG , Viale A , Vidal M , Vieira HLA , Vierstra RD , Vigneswaran N , Vij N , Vila M , Villar M , Villar VH , Villarroya J , Vindis C , Viola G , Viscomi MT , Vitale G , Vogl DT , Voitsekhovskaja OV , von Haefen C , von Schwarzenberg K , Voth DE , Vouret-Craviari V , Vuori K , Vyas JM , Waeber C , Walker CL , Walker MJ , Walter J , Wan L , Wan X , Wang B , Wang C , Wang C-Y , Wang C , Wang C , Wang C , Wang D , Wang F , Wang F , Wang G , Wang H-J , Wang H , Wang H-G , Wang H , Wang H-D , Wang J , Wang J , Wang M , Wang M-Q , Wang P-Y , Wang P , Wang RC , Wang S , Wang T-F , Wang X , Wang X-J , Wang X-W , Wang X , Wang X , Wang Y , Wang Y , Wang Y , Wang Y-J , Wang Y , Wang Y , Wang YT , Wang Y , Wang Z-N , Wappner P , Ward C , Ward DM , Warnes G , Watada H , Watanabe Y , Watase K , Weaver TE , Weekes CD , Wei J , Weide T , Weihl CC , Weindl G , Weis SN , Wen L , Wen X , Wen Y , Westermann B , Weyand CM , White AR , White E , Whitton JL , Whitworth AJ , Wiels J , Wild F , Wildenberg ME , Wileman T , Wilkinson DS , Wilkinson S , Willbold D , Williams C , Williams K , Williamson PR , Winklhofer KF , Witkin SS , Wohlgemuth SE , Wollert T , Wolvetang EJ , Wong E , Wong GW , Wong RW , Wong VKW , Woodcock EA , Wright KL , Wu C , Wu D , Wu GS , Wu J , Wu J , Wu M , Wu M , Wu S , Wu WK , Wu Y , Wu Z , Xavier CP , Xavier RJ , Xia G-X , Xia T , Xia W , Xia Y , Xiao H , Xiao J , Xiao S , Xiao W , Xie C-M , Xie Z , Xie Z , Xilouri M , Xiong Y , Xu C , Xu C , Xu F , Xu H , Xu H , Xu J , Xu J , Xu J , Xu L , Xu X , Xu Y , Xu Y , Xu Z-X , Xu Z , Xue Y , Yamada T , Yamamoto A , Yamanaka K , Yamashina S , Yamashiro S , Yan B , Yan B , Yan X , Yan Z , Yanagi Y , Yang D-S , Yang J-M , Yang L , Yang M , Yang P-M , Yang P , Yang Q , Yang W , Yang WY , Yang X , Yang Y , Yang Y , Yang Z , Yang Z , Yao M-C , Yao PJ , Yao X , Yao Z , Yao Z , Yasui LS , Ye M , Yedvobnick B , Yeganeh B , Yeh ES , Yeyati PL , Yi F , Yi L , Yin X-M , Yip CK , Yoo Y-M , Yoo YH , Yoon S-Y , Yoshida K-I , Yoshimori T , Young KH , Yu H , Yu JJ , Yu J-T , Yu J , Yu L , Yu WH , Yu X-F , Yu Z , Yuan J , Yuan Z-M , Yue BY , Yue J , Yue Z , Zacks DN , Zacksenhaus E , Zaffaroni N , Zaglia T , Zakeri Z , Zecchini V , Zeng J , Zeng M , Zeng Q , Zervos AS , Zhang DD , Zhang F , Zhang G , Zhang G-C , Zhang H , Zhang H , Zhang H , Zhang H , Zhang J , Zhang J , Zhang J , Zhang J , Zhang J-P , Zhang L , Zhang L , Zhang L , Zhang L , Zhang M-Y , Zhang X , Zhang XD , Zhang Y , Zhang Y , Zhang Y , Zhang Y , Zhang Y , Zhao M , Zhao W-L , Zhao X , Zhao YG , Zhao Y , Zhao Y , Zhao Y-X , Zhao Z , Zhao ZJ , Zheng D , Zheng X-L , Zheng X , Zhivotovsky B , Zhong Q , Zhou G-Z , Zhou G , Zhou H , Zhou S-F , Zhou X-J , Zhu H , Zhu H , Zhu W-G , Zhu W , Zhu X-F , Zhu Y , Zhuang S-M , Zhuang X , Ziparo E , Zois CE , Zoladek T , Zong W-X , Zorzano A , Zughaier SM . 2016. Guidelines for the use and interpretation of assays for monitoring autophagy (3rd edition). Autophagy 12:1–222. doi:10.1080/15548627.2015.1100356 26799652 PMC4835977

[39] Takahama M , Akira S , Saitoh T . 2018. Autophagy limits activation of the inflammasomes. Immunol Rev 281:62–73. doi:10.1111/imr.12613 29248000

[40] Rumfield C , Hyseni I , McBride JW , Walker DH , Fang R . 2020. Activation of ASC inflammasome driven by toll-like receptor 4 contributes to host immunity against rickettsial infection. Infect Immun 88:e00886-19. doi:10.1128/IAI.00886-19 32014896 PMC7093143

[41] Kinsella RL , Kimmey JM , Smirnov A , Woodson R , Gaggioli MR , Chavez SM , Kreamalmeyer D , Stallings CL . 2023. Autophagy prevents early proinflammatory responses and neutrophil recruitment during Mycobacterium tuberculosis infection without affecting pathogen burden in macrophages. PLoS Biol 21:e3002159. doi:10.1371/journal.pbio.3002159 37319285 PMC10306192

[42] Ye X , Zhou XJ , Zhang H . 2018. Exploring the role of autophagy-related gene 5 (ATG5) yields important insights into autophagy in autoimmune/autoinflammatory diseases. Front Immunol 9:2334. doi:10.3389/fimmu.2018.02334 30386331 PMC6199349

[43] Moderzynski K , Papp S , Rauch J , Heine L , Kuehl S , Richardt U , Fleischer B , Osterloh A . 2016. CD4+T cells are as protective as CD8+T cells against Rickettsia typhi infection by activating macrophage bactericidal activity. PLoS Negl Trop Dis 10:e0005089. doi:10.1371/journal.pntd.0005089 27875529 PMC5119731

[44] Voss OH , Rahman MS . 2021. Rickettsia-host interaction: strategies of intracytosolic host colonization. Pathog Dis 79:ftab015. doi:10.1093/femspd/ftab015 33705517 PMC8023194

[45] Lin M , Liu H , Xiong Q , Niu H , Cheng Z , Yamamoto A , Rikihisa Y . 2016. Ehrlichia secretes Etf-1 to induce autophagy and capture nutrients for its growth through RAB5 and class III phosphatidylinositol 3-kinase. Autophagy 12:2145–2166. doi:10.1080/15548627.2016.1217369 27541856 PMC5103349

[46] Rikihisa Y . 2017. Role and function of the type IV secretion system in *Anaplasma* and *Ehrlichia* species, p 297–321. In Current topics in microbiology and immunology. Springer. doi:10.1007/978-3-319-75241-9_12 29536364

[47] Rikihisa Y , Lin M . 2010. Anaplasma phagocytophilum and Ehrlichia chaffeensis type IV secretion and Ank proteins. Curr Opin Microbiol 13:59–66. doi:10.1016/j.mib.2009.12.008 20053580 PMC3251840

[48] Rikihisa Y . 2019. Subversion of RAB5-regulated autophagy by the intracellular pathogen Ehrlichia chaffeensis. Small GTPases 10:343–349. doi:10.1080/21541248.2017.1332506 28650718 PMC6748376

[49] Campbell-Valois FX , Sachse M , Sansonetti PJ , Parsot C . 2015. Escape of actively secreting Shigella flexneri from ATG8/LC3-positive vacuoles formed during cell-to-cell spread is facilitated by IcsB and VirA. mBio 6:e02567–14. doi:10.1128/mBio.02567-14 26015503 PMC4447254

[50] Ogawa M , Yoshimori T , Suzuki T , Sagara H , Mizushima N , Sasakawa C . 2005. Escape of intracellular Shigella from autophagy. Science 307:727–731. doi:10.1126/science.1106036 15576571

[51] Kaur SJ , Rahman MS , Ammerman NC , Beier-Sexton M , Ceraul SM , Gillespie JJ , Azad AF . 2012. TolC-dependent secretion of an ankyrin repeat-containing protein of Rickettsia typhi. J Bacteriol 194:4920–4932. doi:10.1128/JB.00793-12 22773786 PMC3430354

[52] Voss OH , Murakami Y , Pena MY , Lee H-N , Tian L , Margulies DH , Street JM , Yuen PST , Qi C-F , Krzewski K , Coligan JE . 2016. Lipopolysaccharide-induced CD300B receptor binding to toll-like receptor 4 alters signaling to drive cytokine responses that enhance septic shock. Immunity 44:1365–1378. doi:10.1016/j.immuni.2016.05.005 27261276 PMC4917413

[53] Yaron S , Matthews KR . 2002. A reverse transcriptase-polymerase chain reaction assay for detection of viable Escherichia coli O157:H7: investigation of specific target genes. J Appl Microbiol 92:633–640. doi:10.1046/j.1365-2672.2002.01563.x 11966903

[54] Klein PG , Juneja VK . 1997. Sensitive detection of viable Listeria monocytogenes by reverse transcription-PCR. Appl Environ Microbiol 63:4441–4448. doi:10.1128/aem.63.11.4441-4448.1997 9361430 PMC168763

[55] Rennoll SA , Rennoll-Bankert KE , Guillotte ML , Lehman SS , Driscoll TP , Beier-Sexton M , Rahman MS , Gillespie JJ , Azad AF . 2018. The cat flea (Ctenocephalides felis) immune deficiency signaling pathway regulates Rickettsia typhi infection. Infect Immun 86:e00562-17. doi:10.1128/IAI.00562-17 29084898 PMC5736803

